# Analysis of allele-specific expression using RNA-seq of the Korean native pig and Landrace reciprocal cross

**DOI:** 10.5713/ajas.19.0097

**Published:** 2019-05-28

**Authors:** Byeongyong Ahn, Min-Kyeung Choi, Joori Yum, In-Cheol Cho, Jin-Hoi Kim, Chankyu Park

**Affiliations:** 1 Department of Stem Cell and Regenerative Biotechnology, Konkuk University, Seoul 05029, Korea; 2 Subtropical Livestock Research Institute, National Institute of Animal Science, Jeju 63242, Korea

**Keywords:** Swine, Allele-specific Expression (ASE), Genomic Imprinting, Reciprocal Cross, Korean Native Pigs

## Abstract

**Objective:**

We tried to analyze allele-specific expression in the pig neocortex using bioinformatic analysis of high-throughput sequencing results from the parental genomes and offspring transcriptomes from reciprocal crosses between Korean Native and Landrace pigs.

**Methods:**

We carried out sequencing of parental genomes and offspring transcriptomes using next generation sequencing. We subsequently carried out genome scale identification of single nucleotide polymorphisms (SNPs) in two different ways using either individual genome mapping or joint genome mapping of the same breed parents that were used for the reciprocal crosses. Using parent-specific SNPs, allele-specifically expressed genes were analyzed.

**Results:**

Because of the low genome coverage (~4×) of the sequencing results, most SNPs were non-informative for parental lineage determination of the expressed alleles in the offspring and were thus excluded from our analysis. Consequently, 436 SNPs covering 336 genes were applicable to measure the imbalanced expression of paternal alleles in the offspring. By calculating the read ratios of parental alleles in the offspring, we identified seven genes showing allele-biased expression (p<0.05) including three previously reported and four newly identified genes in this study.

**Conclusion:**

The newly identified allele-specifically expressing genes in the neocortex of pigs should contribute to improving our knowledge on genomic imprinting in pigs. To our knowledge, this is the first study of allelic imbalance using high throughput analysis of both parental genomes and offspring transcriptomes of the reciprocal cross in outbred animals. Our study also showed the effect of the number of informative animals on the genome level investigation of allele-specific expression using RNA-seq analysis in livestock species.

## INTRODUCTION

Vertical transmission of genetic information in diploid organisms through sexual reproduction ensures an equal amount of genetic contribution from male and female parents for the autosomes. Accordingly, the expression of genetic information from offspring is expected to have an equal presence of maternal and paternal alleles. However, a subset of genes shows deviation from the expected equal presentation of parental alleles and preferentially express the allele from a single parent referred as allele-specific expression (ASE) or allele-biased expression. The degree of expression bias varied from complete monoallelic expression to preferential overexpression of an allele from a single parent [1]. Additionally, the pattern of ASE could be parent-of-origin dependent, namely, genomic imprinting [2] or autosomal random monoallelic expression (RMAE) [3].

The mechanisms underlying imbalanced allelic expression could be several-fold including DNA methylation, histone modification, and the influence of cis- and trans- regulatory elements [4]. The ASE can significantly affect the phenotypes of individual organisms. For example, disruption of the imprinting control elements result in alteration of gene expression and phenotypic abnormalities [5]. Therefore, understanding the nature of ASE associated with epigenetic regulation and identification of loci involved in the phenomenon is important in animal genetics and developmental biology.

Genomic imprinting has been observed in therian species in animals [2]. Approximately more than 180 imprinted genes have been reported in mammals to date and most results were from humans and mice [6]. For livestock species, most studies were of comparative analyses on identified imprinted genes from humans and mice [7]. Approximately 20 genes were confirmed to be imprinted in pigs [6,7]. Therefore, the finding of ASE and subsequent understanding of species variation in livestock species has been limited.

In animal breeding, genomic imprinting could play an im portant role in phenotypes related to economically important traits such as body composition [8]. As an attempt to further understand the mechanisms of epigenetic regulation such as imprinting in pigs, the methylation pattern of the pig genome was analyzed [9,10]. Although many imprinted genes in other species could be still conserved in pigs [7], genome-wide direct investigation of ASE in pigs could significantly contribute to illuminate the characteristics of genomic imprinting in pigs. However, tracing the parent of origin for the expressed genes at the genome level has been a great challenge in outbred animals [11].

The list of genes associated with allelic imbalance in gene expression could be larger than those identified currently. High throughput technologies for genome and transcriptome analyses were successfully employed to better understand ASE at the genome level [12,13]. High-throughput analysis of the neocortex transcriptome from reciprocal crosses of two different strains of mice showed that a much larger number of genes showed differential allelic expression than that expected [12]. A similar study was carried out in pigs without parental genome information [14]; however, studies using both whole genome sequences of parents and the transcriptomes of F_1_ offspring from reciprocal crosses have not been reported in pigs.

In this study, we tried to determine the expression level of each parental allele in RNA-seq analysis results of F_1_ offspring from a pair of reciprocal crosses based on the whole genome sequencing results of parents. We identified nine genes with allele-biased expression, showing both the possibility and limit for the genome-wide identification of genomic imprinting using the reciprocal cross design in outbred animals. Further studies on the newly identified genes of allele-biased expression should expand our current understanding on the ASE in the porcine genome including genomic imprinting.

## MATERIALS AND METHODS

### Animals and sample collection

**Figure 1:**
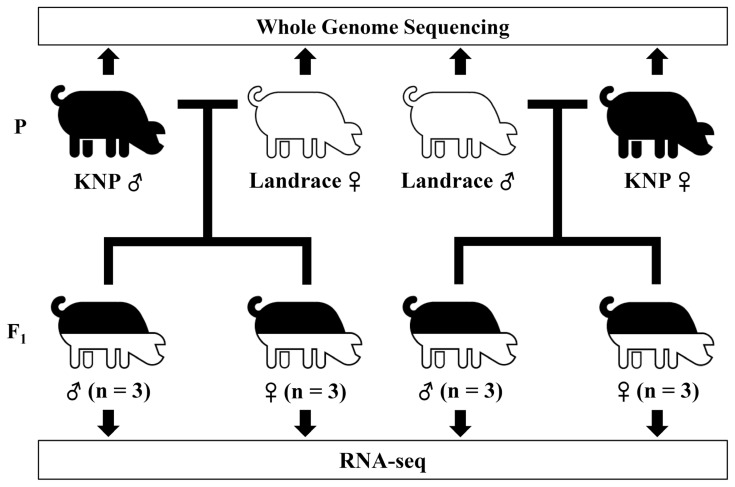
Study design for the evaluation of allele-specific expression from crosses between Korean native and Landrace pigs. Korean native pigs (KNP) and Landrace (Landrace) were reciprocally crossed. Total RNA from three individuals of the same sex from each cross were pooled together and used for RNA-seq.

Four pigs including a male and a female each for Korean native pigs (KNP) and Landrace pigs from populations maintained at the National Institute of Animal Science were selected randomly, and reciprocal crosses were carried out (Figure 1). Fifteen offspring were produced from the crosses. Ear notch tissue samples were collected from the parent animals of the crosses. For the sample collection of the offspring, one-week old piglets were euthanized. Tissues were collected, snap frozen in liquid nitrogen, and stored at −80°C until use. All animal procedures were carried out according to the Institutional Animal Care and Use Committee (IACUC) guidelines of Konkuk University.

### Preparation of genomic DNA and total RNA

Genomic DNA was extracted from 0.5 g of ear tissues as described previously [9]. Briefly, tissues were incubated in lysis buffer (0.1 M Tris-HCl, 200 mM NaCl, 5 mM ethylenediaminetetraacetic acid, 0.2% sodium dodecyl sulfate and 250 μg/mL proteinase K) at 55°C for 6 hours. Subsequently DNA was extracted using phenol extraction and alcohol precipitation. The isolated DNA was treated with DNase-free RNase (Qiagen, Germantown, MD, USA) and further purified using a PowerClean DNA Clean-Up Kit (MO BIO, Carlsbad, CA, USA) according to the manufacturer’s protocol.

Total RNA was extracted from 0.5 g of the neocortex using Trizol (Invitrogen, Carlsbad, CA, USA) according to the manufacturer’s protocol. The isolated RNA was treated with RNase-free DNase (Qiagen, USA). The quality of extracted DNA and RNA was evaluated using a NanoDrop UV/Vis spectrophotometer (Thermo Fisher Scientific, Waltham, MA, USA) and 0.7% agarose gel.

### Next generation sequencing library preparation and sequencing

Construction of next generation sequencing (NGS) libraries and paired-end sequencing using a HiSeq2000 analyzer (Illumina, San Diego, CA, USA) was performed at BGI-Shenzen (Shenzhen, China). NGS libraries were constructed with one microgram of genomic DNA using the TruSeq DNA sample prep kit (Illumina, USA) according to the manufacturer’s protocol. Construction of RNA-seq libraries and single-end sequencing using a HiSeq2000 analyzer (Illumina, USA) was performed at DNA link (Seoul, Korea). An equal amount of total RNA from three offspring of the same sex in each cross were pooled. RNA-seq libraries were constructed with one microgram of high-quality RNA using a TruSeq Stranded Total RNA Library Prep Kit (Illumina, USA) according to the manufacturer’s protocol.

### Read mapping

A pig reference genome assembly Sscrofa11.1 (GenBank accession: GCA_000003025.6) was downloaded from Ensembl (release 92). Sequencing reads were aligned to the reference using the BWA MEM package (version 0.7.17-r1188) [15]. SAM files were converted to the BAM format using samtools index, and were sorted using samtools sort [16]. Mapping of RNA-seq reads to the reference genome was carried out using the STAR package (version 2.5.3a) with the default options and 2-pass mode [17].

### Variant calling and filtration

Polymerase chain reaction duplicates of the mapped reads were removed using MarkDuplicates in Picard tools (version 2.15.0, https://broadinstitute.github.io/picard/). For RNA-seq reads, the read group was added to the mapped reads using GATK AddOrReplaceReadGroups, and overhanging reads mapped in intronic regions were removed using GATK Split NCigarReads [18]. Base quality of the reads was recalibrated using GATK BaseRecalibrator (version 3.8) for both whole genome sequencing and RNA-seq results, and the quality-adjusted reads were obtained using GATK PrintReads. Variants of parental genomes and offspring transcriptomes were called using GATK and HaplotypeCaller, respectively, and the called variants were joint-genotyped using GATK GenotypeGVCFs [18]. The genotyped variants were annotated with NCBI dbSNP 150 [19] and ENSEMBL annotation (release 92) using GATK VariantAnnotator and snpEff [20]. Subsequently, variants with strong strand bias (Fisher strand>30), low quality depth (<2) and single nucleotide polymorphism (SNP) clusters where 3 or more SNPs are located within a 35 bp window were removed using GATK VariantFiltration and SelectVariants. We also filtered out low depth variants (read depth [DP] <3 for individual mapping and DP<6 for the same breed joint mapping). Finally, exonic SNPs were selected using GATK SelectVariants and snpSift [21].

### Discovery of allele-biased expression and genomic imprinting

**Figure 2:**
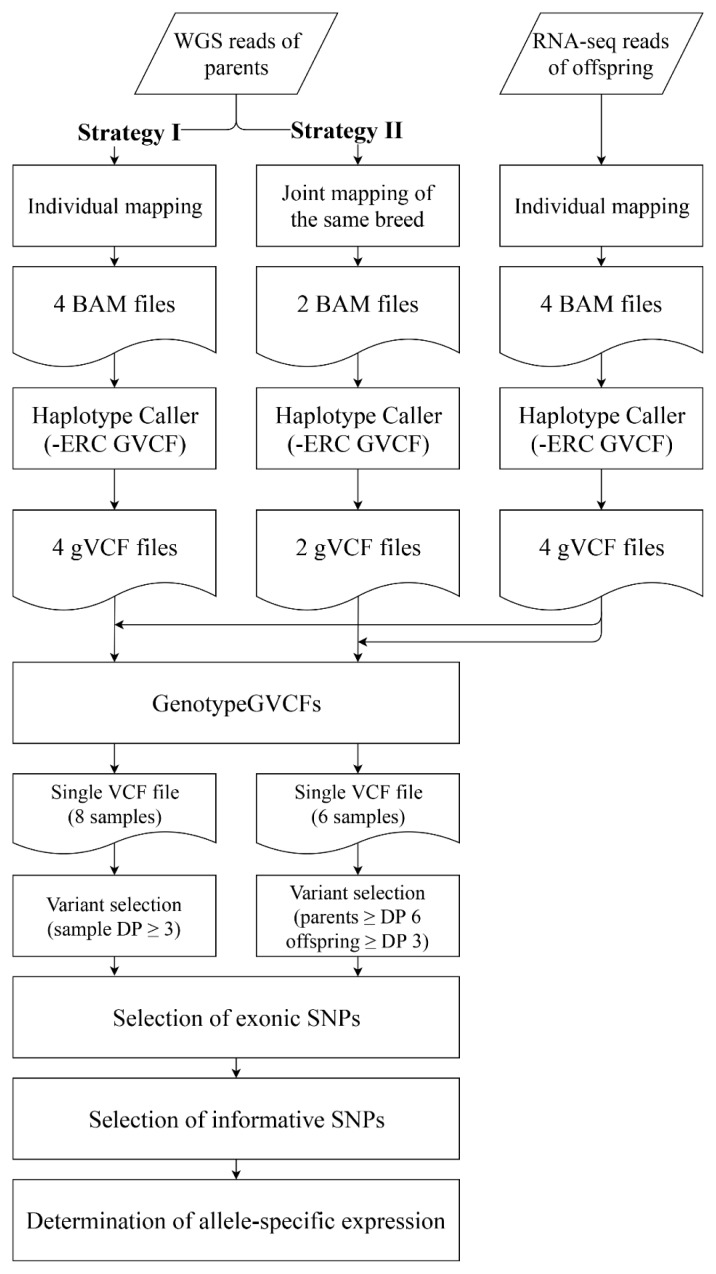
Workflow of bioinformatic analysis to identify candidate single nucleotide polymorphisms for the discovery of allele-specific expression. Strategies I and II differ in mapping parental reads. DP, depth of reads mapped to the position.

The steps of ASE identification consisted of the selection of exonic and informative SNPs, and subsequent determination of ASE (Figure 2). We selected SNPs which are homozygous in each parent but differ between male and female parents as informative SNPs to distinguish the origin of SNPs and the level of relative expression. To determine genes showing deviated expression at a 1:1 ratio between paternal and maternal alleles, the paternal read ratio for selected candidate genes with informative SNPs was calculated from RNA-seq results using the following equation,

$$Paternal\;read\;ratio=\frac{paternal\;read\;counts}{maternal\;read\;counts+paternal\;read\;counts}$$

The ratio of maternal reads was calculated as 1– *paternal read ratio*. The adopted arbitrary criteria to determine bias in allelic expression in our study was either <0.3 or >0.7 for any given allele. When the ratio was between 0.3 and 0.7, we considered it as biallelic expression. The G-test for goodness-of-fit was used to determine statistical significance [22].

## RESULTS

### Sequence analysis of parental genomes and the neocortex transcriptome of the offspring

Determination of ASE in offspring neocortex requires identification of the parent of origin for the expressed alleles. Therefore, we performed whole genome sequencing for four parents constituting the reciprocal crosses between KNP and Landrace pigs and obtained the whole genome sequencing results of 111 to 117 million paired-end reads with 90 bp in length for each parent (Table 1). The genome coverage and mapping rates against the current reference pig genome assembly ranged from 4.05 to 4.25× and 99.45% to 99.42%, respectively, indicating that most of the pig genome was covered. In addition, we also carried out joint mapping of sequencing reads for two KNP or two Landrace pigs, respectively, to increase the read depth, achieving 8.19 and 8.27× coverage for KNP and Landrace, respectively, which could increase the number of identified breed- or parent-specific SNPs.

To identify genes showing allele-biased expression in offspring, RNA-seq analysis was carried out using pooled RNA of the neocortex from three offspring of the same sex for each reciprocal cross. Thus, we obtained 10.08 to 12.51 million RNA-seq reads from four different samples (Table 1). The mapping rates against the current pig gene annotation ranged from 98.56% to 98.86% and the read depth to exonic regions ranged from 12× to 15×.

### Identification of nucleotide variants from Korean native and Landrace pigs

Our strategy to identify genes with ASE is described in Figure 2. Because the animals used in this study were not inbred with identity by descent (IBD), our analysis was limited to loci meeting the condition of intra-breed homozygosity and inter-breed allelic difference for KNP and Landrace to distinguish segregation from parents to offspring. Furthermore, only exonic variants were informative to identify genes with ASE.

We used two different strategies to map whole genome sequencing reads of parents constituting our reciprocal crosses to determine the parental origin of expressed alleles for given genes (Figure 2). The first (strategy I) was to individually map the genome sequencing results of each parent to the reference genome, resulting in a total of four alignment files, one for each parent. The second (strategy II) was joint mapping of whole genome sequencing results of the same breed (KNP or Landrace) to increase the number of informative SNPs for ASE determination from low depth sequencing results, resulting in two alignment files, one each for KNP and Landrace.

The alignment files generated in two different ways were analyzed together with four alignment files generated from the neocortex RNA-seq read mapping of the offspring for variant calling (Table 2). The total number of identified SNPs from the initial raw variants satisfying our filtration criteria (see methods) except for read depth were 10,005,109 and 9,609,853 for strategies I and II, respectively, which are similar to the number of SNPs segregated among four Asian wild boars (11,472,192) in a demographic study of pig genomes [23]. We then removed the variants with low confidence and mapped to noncoding regions, retaining only 11,683 and 26,809 variants for each strategy. Additional analysis to select exonic SNPs resulted in only 7,998 and 18,065 SNPs, which represent 5,602 and 8,111 genes, respectively.

### Selection of single nucleotide polymorphisms applicable to determine allele-specific expression

**Figure 3:**
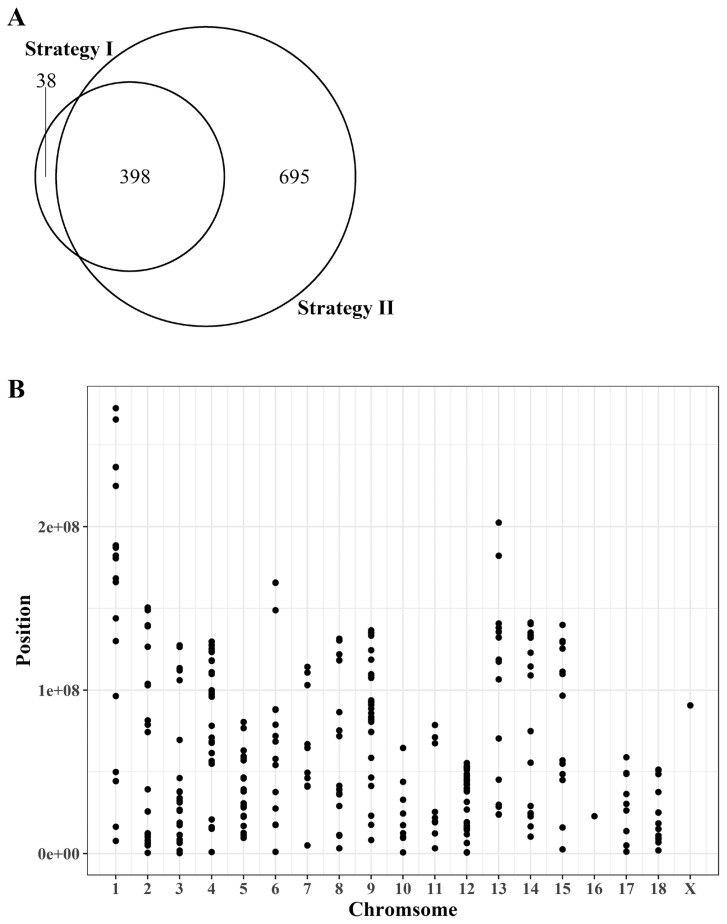
Distribution of the identification of informative SNPs using two different mapping strategies. (A) Number of overlapped and unique SNPs identified to evaluate allele-biased expression from two different mapping strategies. (B) Distribution of informative SNPs from strategy I. SNPs, single nucleotide polymorphisms.

Our analysis to identify genes showing ASE under the criteria of RNA-seq read counts of <30% or >70% for any given allele resulted in identification of 436 and 1,093 candidate SNPs according to two different strategies, respectively (Figure 3A, Supplementary Table S1). Among them, 398 were present in both strategies and 38 and 695 SNPs were unique for each strategy. This indicates that the results are somewhat different depending on the mapping strategies of sequencing reads. Because strategy I contains a lower number of unique SNPs compared to strategy II, the result of strategy I was subjected to further analysis for evaluation of allele-biased expression. Because identified SNPs from strategy I contain a higher number of common SNPs with those of strategy II showing a large number of strategy specific SNPs, the SNPs identified from strategy I was subjected to evaluate the presence of allele-biased expression while minimizing the possibility to identify false positive ASE. The 436 identified SNPs from the strategy I were evenly distributed across genomes except for chromosome 16 and the sex chromosomes (Figure 3B). Because of the limit in the number of breed-specific SNPs applicable for quantification of allelic bias in the expression of parental alleles, our analysis was limited to testing only 336 genes for their allelic imbalance rather than a genome-wide evaluation.

### Identification of nine genes showing allele-specific expression in the neocortex of pigs

We analyzed the presence of imbalance in the allelic expression of genes associated with 436 SNPs in the neocortex transcriptome of the offspring of KNP×Landrace reciprocal crosses. RNA-seq analysis revealed that SNPs corresponding to 7 genes including nucleolar and spindle associated protein 1 (*NUSAP1*), family with sequence similarity 83 member H (*FAM83H*), solute carrier family 6 member 17 (*SLC6A17*), mannosidase beta (*MANBA*), paternally expressed 10 (*PEG10*), ENSSSCG 00000010703, and ENSSSCG00000010719 showed allele-biased expression (p<0.05, Table 3, Supplementary Table S1). In addition, transferrin receptor 2 (*TFR2*) and PPFIA binding protein 1 (*PPFIBP1*) also seem to be allele-specifically expressed but their p-values were not significant. Especially, *NUSAP1* and *PEG10* showed extreme expression biases toward paternal alleles. *PPFIBP1* showed maternal allele-biased expression. In addition, *FAM83H*, *SLC6A17*, *MANBA*, *TFR2*, ENSSSCG00000010703, and ENSSSCG00000010719 showed dominant expression of a specific allele without influence of the origin of parent. In the case of *NUSAP1*, *PEG10*, and *PPFIBP1*, the genes showed a flipped allelic expression pattern in which the same allele shows the opposite expression pattern depending on the origin of the parent between a pair of reciprocal crosses, indicating a strong evidence of genomic imprinting.

## DISCUSSION

Analysis of genes showing ASE using the reciprocal cross of inbred animals is an effective method to discover genomic imprinting associated genes [12,13]. However, the use of similar approaches for outbred animals like pigs is challenging because of inherent difficulty in distinguishing the parental origin of any given allele due to the presence of segregating multi allelic polymorphisms in the breed [11]. To investigate the efficiency of experimental outcomes for detecting ASE from the reciprocal cross design in outbred animals and newly identify those genes, we carried out a pair of reciprocal crosses using KNP and Landrace pigs, determined the parental lineage of alleles, and analyzed the presence of ASE in genes from F1 animals. Because of the low-depth read coverage of parental genomes (~4×) and transcriptomes (~15×) of offspring, genome-wide evaluation of biased allelic expression was not achieved. However, we were able to present several genes showing allele-biased expression including a well-known imprinted gene, *PEG10*. We also compared the efficiency of two different read mapping strategies for the bioinformatic determination of ASE at a low-depth read coverage in outbred animals.

Discovery of the flipped allelic expression pattern at SNP positions from F1 animals of the reciprocal crosses can suggest the presence of allele-biased expression patterns such as genomic imprinting. However, the heterozygous SNP positions are not always informative concerning transmission in outbred strains or lines, and even not all SNP positions are heterozygous. Therefore, determination of parent of origin for a given allele is often unresolvable, which leads to significant restriction in genetic analyses. It has been suggested that a large sample size (at least >30 informative individuals) is necessary for efficient evaluation of allele-biased expression using RNA-seq for outbred or semi-inbred species to achieve genome-level coverage [24].

In this study, we analyzed four neocortex transcriptomes consisting of pooled RNA from three individuals for each library using 12 F_1_ animals from KNP×Landrace reciprocal crosses to reduce the number of RNA-seq analyses. However, the lower read depth for mapped genes in our sequencing results does not allow us to clearly determine the origin of parents in the offspring. Thus, we are only able to use the variant information in homozygous status to estimate allele-biased expression. Consequently, only a limited number of genes were evaluated in our results despite the use of whole genome sequences of parents. Our results also suggest that the use of individual sequencing strategies is likely to provide improved results compared to the analysis of pooled samples.

Determination of parental origin of expressed alleles in F_1_ individuals from RNA-seq data can be efficiently achieved using bioinformatic analysis tools if parent-specific SNPs are clearly distinguishable. However, variant calling in RNA-seq is still challenging because of experimental limitations such as biases from library preparation, low sequencing read depth, experimental errors, and biological variations such as ASE, splicing variation, and RNA editing [25]. Therefore, the results of variant calling may significantly differ depending on the analysis tools and statistical values.

To overcome the disadvantage of low sequencing depth, we carried out bioinformatic analysis in two different ways by either mapping the genome sequencing results of each parent individually or of two parents of the same breed together to determine the breed- or parent-specific SNPs. The joint read mapping showed about two-fold increase in the number of candidate SNPs available for evaluating allele-biased expression, but the increase was still limited (Figure 3A), suggesting that the number of informative individuals is critical for genome wide analysis in outbred animals. However, we also noticed unique SNPs associated with each strategy (Figure 3A). The difference could be due to a bias in SNP calling from the difference in read depth between the two strategies.

To understand the difference between the strategies, we carried out manual confirmation of the identified candidate SNPs using raw variant data files. Most conflicts in SNP calling either produced false-positive SNPs from homozygotes or failure in detecting SNPs from heterozygotes due to the low read depth (data not shown). However, the error rate was lower in strategy I and results were more consistent compared to those of strategy II which involved joint mapping of two parents of the same breed.

We identified nine allele-biased-expressed genes in the neocortex of pigs using the described bioinformatic procedure in Table 3. Among them, *PEG10* is a known paternally imprinted gene in both human and pig [7], and this gene has been reported to be associated with several malignancies, such as hepatocellular carcinoma and B-cell lymphocytic leukemia in human [26]. ASE of *SLC6A17* and *MANBA* has also been reported in previous studies investigating other species [13,27]. The protein encoded by *SLC6A17* is a member of the SLC6 family of transporters, which are responsible for the presynaptic uptake of neurotransmitters [28]. *MANBA* encodes beta-mannosidase which localizes to the lysosome [29]. Three out of seven genes (43%) that we observed to show allele-biased expression in this study were reported previously, indicating that the bioinformatic strategy used in this study is suitable for identifying allele-biased expression in outbred strains.

Although further experimental confirmation remains to be carried out to clearly prove the ASE through independent breeding experiments, we suggested a list of new candidate genes for the ASE in pigs. However, the number of animals used for reciprocal crosses and sequencing read depth should be increased to cover a large number of genes as the genome wide analysis.

*NUSAP1* is a nucleolar-spindle-associated protein that plays a role in spindle microtubule organization [30]. However, no information has been available regarding its ASE. The expression pattern of *FAM83H*, *SLC6A17*, *MANBA*, ENSSSCG 00000010703, and ENSSSCG00000010719 was different from that of genomic imprinting, which could be explained by cis-regulating expression quantitative trait loci [31] or RMAE [3]. In addition, although statistically less significant, *PPFIBP1*, which encodes liprin-beta-1 protein acting functioning in cell adhesion [32], showed an expression pattern of maternal imprinting (Table 3, Supplementary Table S1).

Taken together, our results showed that the strategy and bioinformatics pipeline used in this study are suitable for the identification of genes showing allele-biased expression from reciprocal crosses of outbred animals with some limitations. Experimental validation of candidate genes and further studies on these genes should provide new information on genomic imprinting in pigs.

## Supplementary Data



## Figures and Tables

**Table 1:** General statistics of genome and transcriptome sequencing and mapping

Items	Raw reads (M)	Mapping rate (%)	Mapped bases (G)	Coverage (×)1)
WGS (parents)				
KNP ♂	114.67	99.34	10.19	4.15
KNP ♀	112.08	99.38	9.95	4.05
Landrace ♂	111	99.45	9.87	4.02
Landrace ♀	117.5	99.42	10.45	4.25
KNP combined	226.75	99.36	20.14	8.19
Landrace combined	228.5	99.43	20.32	8.27
RNA-seq2) (offspring)				
L×K ♂	10.08	98.56	0.91	12.59
L×K ♀	12.51	98.79	1.14	15.77
K×L ♂	14.96	98.86	1.37	18.96
K×L ♀	12.02	98.84	1.1	15.22

WGS, whole genome sequencing; KNP, Korean native pigs; L×K, Landrace×Korean native pig; K×L, Korean native pig×Landrace; ♂, male; ♀, female.

1) The coverage of WGS and RNA-seq corresponds to that of the pig genome and the annotated protein coding region, respectively.

2) RNA-seq was carried out using the pooled total RNA of three individuals.

**Table 2:** Number of variants identified from two different mapping strategies

Items	Individual mapping (Strategy I)	Joint mapping of two individuals of the same breed (Strategy II)
Raw variants	16,799,276	16,722,260
Filtered variants (SNP+INDEL)	11,683	26,809
SNP	10,444	23,749
Exonic SNP	7,998	18,065

SNP, single nucleotide polymorphism; INDEL, insertion or deletion.

**Table 3:** List of genes showing allele-specific expression

Chr.	Position	Gene	Paternal-allele read ratio1)			

			K×L cross		L×K cross	
	
			♂	♀	♂	♀
1	129993052	NUSAP1*	1	1	1	1
4	907296	FAM83H2*	1	1	0	0
4	109921555	SLC6A17*	0.897	0.667	0.094	0.329
8	118361691	MANBA2*	0	0	1	1
9	74485347	PEG10*	1	1	1	1
14	132103321	ENSSSCG00000010703*	0.667	0.765	0.282	0.188
14	132495049	ENSSSCG00000010719*	0.778	0.92	0.174	0.2
3	8560671	*TFR2*	0.222	0	0.9	0.727
5	46032153	*PPFIBP*1	0.286	0.235	0	0.3

Chr., chromosomes, “K” and “L” indicate Korean native pigs and Landrace, respectively.

*NUSAP1*, nucleolar and spindle associated protein 1; *FAM83H*, family with sequence similarity 83 member H; *SLC6A17*, solute carrier family 6 member 17; *MANBA*, mannosidase beta; *PEG10*, paternally expressed 10; *TFR2*, transferrin receptor 2; *PPFIBP1*, PPFIA binding protein.

1) The symbols ♂ (males) and ♀ (females) indicate the sex of the offspring used for RNA-seq analysis.

* Indicates the statistical significance (p<0.05) on the unequal expression of maternal and paternal alleles.
