# Supplementary material for: Analysis of allele-specific expression using RNA-seq of the Korean native pig and Landrace reciprocal cross
Source: Asian-Australas J Anim Sci. 2019 May 28;32(12):1816–25. doi: 10.5713/ajas.19.0097 (PMC6819674; doi:10.5713/ajas.19.0097)
Supplement: Supplementary file 1 [file ajas-19-0097-suppl.pdf]

Table S1. Identified candidate SNPs from strategy I

This table contains the total candidate SNPs which can be used to infer allele-specific expression.
The level of expression of a paternal allele was colored blue, and that of maternal allele red for better visualization.

Abbreviation	Description
CHR	chromosome
POS	position
GT	Genotype
AD	Allelic Depth (# of reference allele, # of alteriative allele
KL	F1 from cross between KNP male and Landrace female
LK	F1 from cross between Landrace male and KNP female

CHR	POS	ID	REF	ALT	Gene_name	Paternal reads ratio				Significance	G-test_P-value				GT and AD of F1 offspring								GT and AD of the parents							
						KL ♂	KL ♀	LK ♂	LK ♀		KL ♂	KL ♀	LK ♂	LK ♀	KL ♀ GT	KL ♀ AD	KL ♂ GT	KL ♂ AD	LK ♀ GT	LK ♀ AD	LK ♂ GT	LK ♂ AD	KNP ♀ GT	KNP ♀ AD	KNP ♂ GT	KNP ♂ AD	Landrace ♀ GT	Landrace ♀ AD	Landrace ♂ GT	Landrace ♂ AD
1	7689256	rs80848798	G	T	SOD2	0.440	0.571	0.286	0.500	FALSE	0.548	0.512	0.249	1.000	G/T	12,9	G/T	11,14	G/T	9,9	G/T	5,2	G/G	6,0	G/G	6,0	T/T	0,6	T/T	0,6
1	7689419	rs330195282	T	A	SOD2	0.514	0.500	0.667	0.476	FALSE	0.866	1.000	0.192	0.827	T/A	12,12	T/A	18,17	T/A	11,10	T/A	5,10	T/T	3,0	T/T	5,0	A/A	0,3	A/A	0,5
1	7691513	.	T	G	SOD2	0.467	0.393	0.471	0.425	FALSE	0.715	0.255	0.732	0.342	T/G	11,17	T/G	14,16	T/G	23,17	T/G	18,16	T/T	7,0	T/T	4,0	G/G	0,8	G/G	0,7
1	16362583	rs332236820	C	A	GINM1	0.429	0.000	0.714	0.571	FALSE	0.705	0.001	0.249	0.705	A/A	0,8	C/A	3,4	C/A	3,4	C/A	2,5	C/C	4,0	C/C	8,0	A/A	0,3	A/A	0,7
1	16362878	rs55618829	T	C	GINM1	0.231	0.200	0.714	0.000	FALSE	0.046	0.050	0.249	0.019	T/C	2,8	T/C	3,10	T/T	4,0	T/C	2,5	T/T	5,0	T/T	3,0	C/C	0,3	C/C	0,7
1	44285697	rs324450373	A	T	ENSSSCG00000004253	0.300	0.000	0.727	0.333	FALSE	0.200	0.000	0.125	0.313	T/T	0,10	A/T	3,7	A/T	6,3	A/T	3,8	A/A	3,0	A/A	5,0	T/T	0,5	T/T	0,6
1	49932001	rs344386379	G	C	LMBRD1	0.304	0.500	0.000	0.571	FALSE	0.057	1.000	0.041	0.705	G/C	8,8	G/C	7,16	G/C	3,4	G/G	3,0	G/G	6,0	G/G	5,0	C/C	0,3	C/C	0,5
1	96442468	rs1111495896	C	T	PIAS2	0.524	0.800	1.000	0.750	FALSE	0.827	0.050	0.041	0.076	C/T	2,8	C/T	10,11	C/T	9,3	C/C	3,0	T/T	0,3	T/T	0,4	C/C	5,0	C/C	5,0
1	96442822	rs1111904365	C	G	PIAS2	0.571	0.400	0.000	0.750	FALSE	0.592	0.526	0.041	0.148	C/G	6,4	C/G	6,8	C/G	6,2	G/G	0,3	G/G	0,5	G/G	0,3	C/C	3,0	C/C	3,0
1	129993052	rs322512685	C	G	NUSAP1	1.000	1.000	1.000	1.000	TRUE	0.002	0.041	0.041	0.004	C/C	3,0	C/C	7,0	C/C	6,0	C/C	3,0	G/G	0,3	C/C	4,0	G/G	0,4	C/C	9,0
1	143988103	rs337382926	G	A	MPHOSPH10	0.333	0.857	0.000	0.714	FALSE	0.313	0.047	0.008	0.249	G/A	6,1	G/A	3,6	G/A	2,5	G/G	5,0	G/G	5,0	G/G	9,0	A/A	0,4	A/A	0,3
1	166108051	rs343859810	A	G	CLN6	1.000	1.000	0.833	0.619	FALSE	0.000	0.000	0.001	0.273	G/G	0,9	G/G	0,12	A/G	8,13	A/G	4,20	A/A	3,0	G/G	0,7	A/A	4,0	G/G	0,6
1	166108066	rs323953237	G	A	CLN6	0.923	1.000	0.810	0.609	FALSE	0.001	0.001	0.003	0.295	A/A	0,8	A/A	1,12	G/A	9,14	G/A	4,17	G/G	3,0	A/A	0,7	G/G	3,0	A/A	0,6
1	168365407	rs342129746	T	C	UACA	0.000	0.571	0.000	0.375	FALSE	0.004	0.592	0.041	0.477	T/C	6,8	T/T	6,0	T/C	5,3	T/T	3,0	T/T	6,0	C/C	0,3	T/T	4,0	C/C	0,7
1	180723851	rs80993261	A	G	TRIM9	0.526	0.563	0.451	0.544	FALSE	0.691	0.386	0.483	0.508	A/G	21,27	A/G	27,30	A/G	26,31	A/G	28,23	A/A	4,0	G/G	0,4	A/A	4,0	G/G	0,4
1	182261086	rs323757257	G	C	GPR137C	0.000	0.000	1.000	0.667	FALSE	0.000	0.004	0.004	0.410	G/G	6,0	G/G	15,0	G/C	4,2	G/G	6,0	C/C	0,3	C/C	0,3	G/G	7,0	G/G	3,0
1	182265955	rs328776656	A	G	ERO1A	0.500	0.583	0.471	0.500	FALSE	1.000	0.413	0.808	1.000	A/G	14,10	A/G	12,12	A/G	11,11	A/G	9,8	A/A	3,0	A/A	4,0	G/G	0,5	G/G	0,5
1	187118755	rs343422825	C	T	C14orf37	0.444	0.552	0.360	0.313	FALSE	0.563	0.577	0.159	0.032	C/T	16,13	C/T	12,15	C/T	22,10	C/T	16,9	C/C	6,0	C/C	3,0	T/T	0,3	T/T	0,4
1	187452344	rs330523244	A	G	ARID4A	0.455	0.625	0.615	0.455	FALSE	0.763	0.477	0.403	0.763	A/G	5,3	A/G	5,6	A/G	6,5	A/G	5,8	A/A	9,0	A/A	3,0	G/G	0,5	G/G	0,5
1	188617025	rs324189922	A	G	JKAMP	0.500	0.529	1.000	0.357	FALSE	1.000	0.808	0.019	0.282	A/G	8,9	A/G	6,6	A/G	5,9	A/A	4,0	G/G	0,4	G/G	0,3	A/A	5,0	A/A	6,0
1	224961323	rs330196137	G	A	TMEM2	0.600	0.667	0.818	0.667	FALSE	0.526	0.410	0.028	0.244	G/A	4,2	G/A	6,4	G/A	4,8	G/A	2,9	G/G	4,0	G/G	4,0	A/A	0,5	A/A	0,3
1	236316793	rs80842018	A	G	RUSC2	0.485	0.529	0.625	0.545	FALSE	0.862	0.732	0.477	0.670	A/G	16,18	A/G	17,16	A/G	12,10	A/G	5,3	G/G	0,3	G/G	0,3	A/A	4,0	A/A	4,0
1	236524904	rs321958529	T	C	NPR2	0.500	0.563	0.571	0.273	FALSE	1.000	0.617	0.705	0.125	T/C	7,9	T/C	7,7	T/C	3,8	T/C	4,3	C/C	0,3	C/C	0,3	T/T	3,0	T/T	4,0
1	236529057	rs321698712	C	T	NPR2	0.455	0.571	0.458	0.357	FALSE	0.763	0.705	0.683	0.282	C/T	3,4	C/T	6,5	C/T	5,9	C/T	11,13	T/T	0,7	T/T	0,4	C/C	5,0	C/C	12,0
1	236530335	rs322042520	A	G	SPAG8	0.647	0.000	0.429	0.667	FALSE	0.222	0.004	0.592	0.153	A/A	6,0	A/G	6,11	A/G	12,6	A/G	6,8	G/G	0,5	G/G	0,6	A/A	5,0	A/A	4,0
1	265604270	rs337946665	G	T	OLFML2A	0.000	0.000	0.778	0.500	FALSE	0.041	0.008	0.086	1.000	G/G	5,0	G/G	3,0	G/T	5,5	G/T	7,2	T/T	0,4	T/T	0,4	G/G	6,0	G/G	3,0
1	265637081	rs341480864	T	C	WDR38	0.125	0.091	0.231	0.750	FALSE	0.001	0.003	0.046	0.148	T/C	1,10	T/C	2,14	T/C	2,6	T/C	10,3	T/T	3,0	T/T	3,0	C/C	0,8	C/C	0,5
1	272479160	rs334669976	A	G	GTF3C4	0.273	0.571	1.000	0.286	FALSE	0.125	0.705	0.019	0.249	A/G	3,4	A/G	8,3	A/G	2,5	A/A	4,0	G/G	0,3	G/G	0,3	A/A	4,0	A/A	6,0
1	272479228	rs342961790	G	A	GTF3C4	0.357	0.444	0.250	0.583	FALSE	0.282	0.739	0.306	0.563	G/A	5,4	G/A	9,5	G/A	7,5	G/A	1,3	A/A	0,4	A/A	0,5	G/G	6,0	G/G	4,0
2	549973	rs337416916	C	T	TSPAN4	0.529	0.571	0.444	0.500	FALSE	0.808	0.592	0.637	1.000	C/T	8,6	C/T	9,8	C/T	8,8	C/T	10,8	C/C	6,0	C/C	6,0	T/T	0,6	T/T	0,4
2	550461	rs337530058	A	C	CHID1	0.400	1.000	0.455	0.444	FALSE	0.654	0.041	0.763	0.739	A/A	3,0	A/C	2,3	A/C	5,4	A/C	6,5	A/A	4,0	A/A	4,0	C/C	0,8	C/C	0,3
2	4952329	rs335413914	C	A	NUDT8	0.250	0.500	1.000	0.500	FALSE	0.076	1.000	0.002	1.000	C/A	3,3	C/A	9,3	C/A	4,4	C/C	7,0	A/A	0,4	A/A	0,4	C/C	3,0	C/C	5,0
2	4952334	rs344721689	T	C	NUDT8	0.308	0.625	1.000	0.375	FALSE	0.160	0.477	0.002	0.477	T/C	3,5	T/C	9,4	T/C	3,5	T/T	7,0	C/C	0,4	C/C	0,4	T/T	3,0	T/T	5,0
2	6121064	rs332617373	T	C	KLC2	0.542	0.412	0.643	0.571	FALSE	0.563	0.302	0.128	0.449	T/C	14,20	T/C	26,22	T/C	12,16	T/C	10,18	T/T	4,0	T/T	6,0	C/C	0,3	C/C	0,4
2	7911775	rs334192644	C	A	STIP1	0.417	0.568	0.590	0.368	FALSE	0.247	0.410	0.261	0.028	C/A	16,21	C/A	28,20	C/A	25,43	C/A	23,16	A/A	0,5	A/A	0,5	C/C	3,0	C/C	4,0
2	10009632	rs329433797	A	G	SYT7	0.524	0.583	1.000	0.286	FALSE	0.827	0.563	0.008	0.249	A/G	7,5	A/G	11,10	A/G	5,2	G/G	0,5	A/A	4,0	A/A	4,0	G/G	0,3	G/G	0,3
2	11693986	rs322834518	G	A	MRPL16	0.727	0.875	0.500	0.652	FALSE	0.125	0.024	1.000	0.141	G/A	1,7	G/A	3,8	G/A	15,8	G/A	10,10	A/A	0,7	A/A	0,7	G/G	3,0	G/G	5,0
2	12496627	rs326787208	T	G	ENSSSCG00000013155	0.417	0.526	0.500	0.379	FALSE	0.413	0.819	1.000	0.191	T/G	10,9	T/G	10,14	T/G	18,11	T/G	5,5	T/T	4,0	T/T	3,0	G/G	0,5	G/G	0,9
2	25714538	rs325242142	G	A	SLC1A2	0.396	0.429	0.000	0.667	FALSE	0.129	0.449	0.041	0.313	G/A	12,16	G/A	21,32	G/A	3,6	G/G	3,0	G/G	5,0	G/G	5,0	A/A	0,4	A/A	0,3
2	25763206	rs331807211	G	A	SLC1A2	0.453	0.524	0.333	0.200	FALSE	0.453	0.758	0.560	0.165	G/A	20,22	G/A	35,29	G/A	1,4	G/A	1,2	A/A	0,3	A/A	0,6	G/G	5,0	G/G	10,0
2	25763245	rs339200216	A	G	SLC1A2	0.438	0.487	0.429	0.250	FALSE	0.317	0.873	0.705	0.306	A/G	20,19	A/G	36,28	A/G	1,3	A/G	3,4	G/G	0,3	G/G	0,6	A/A	3,0	A/A	5,0
2	39265586	rs345617015	G	A	NAV2	0.750	0.625	0.857	0.571	FALSE	0.076	0.477	0.047	0.705	G/A	5,3	G/A	9,3	G/A	3,4	G/A	1,6	G/G	4,0	G/G	4,0	A/A	0,3	A/A	0,7
2	74381086	rs81474157	T	C	CHAF1A	0.500	0.000	0.400	0.250	FALSE	1.000	0.041	0.654	0.076	T/T	3,0	T/C	2,2	T/C	9,3	T/C	3,2	T/T	4,0	C/C	0,3	T/T	3,0	C/C	0,6
2	78879756	rs328388044	C	G	CANX	0.500	0.500	0.712	0.712	FALSE	1.000	1.000	0.000	0.000	C/G	28,28	C/G	28,28	C/G	21,52	C/G	19,47	C/C	3,0	C/C	4,0	G/G	0,6	G/G	0,3
2	81540468	rs81211192	T	C	SIMC1	0.500	0.000	0.667	0.000	FALSE	1.000	0.008	0.560	0.008	T/T	5,0	T/C	2,2	T/T	5,0	T/C	1,2	T/T	4,0	C/C	0,3	T/T	3,0	C/C	0,3
2	103005885	.	A	G	PCSK1	0.471	0.467	0.450	0.571	FALSE	0.674	0.655	0.654	0.449	A/G	21,24	A/G	24,27	A/G	12,16	A/G	11,9	A/A	4,0	A/A	3,0	G/G	0,5	G/G	0,5
2	103378438	rs342931877	A	G	CAST	0.455	0.800	0.615	0.556	FALSE	0.763	0.050	0.403	0.739	A/G	8,2	A/G	5,6	A/G	4,5	A/G	5,8	A/A	10,0	A/A	3,0	G/G	0,4	G/G	0,7
2	103801675	.	G	A	ENSSSCG00000036529	0.714	0.500	0.000	0.000	FALSE	0.249	1.000	0.019	0.041	G/A	3,3	G/A	5,2	G/G	3,0	G/G	4,0	G/G	3,0	G/G	3,0	A/A	0,5	A/A	0,9
2	126507663	rs342970004	G	C	SNX24	0.438	0.381	0.368	0.471	FALSE	0.617	0.273	0.249	0.808	G/C	13,8	G/C	9,7	G/C	8,9	G/C	7,12	C/C	0,4	C/C	0,7	G/G	3,0	G/G	4,0
2	126507736	rs328624428	A	G	SNX24	0.667	0.400	0.625	0.591	FALSE	0.192	0.369	0.218	0.392	A/G	12,8	A/G	5,10	A/G	13,9	A/G	15,9	G/G	0,3	G/G	0,4	A/A	3,0	A/A	5,0

2	139020570	rs344956600	G	A	SPOCK1	0.412	0.385	0.250	0.500	FALSE	0.466	0.237	0.148	1.000	G/A	10,16	G/A	7,10	G/A	7,7	G/A	6,2	G/G	5,0	G/G	3,0	A/A	0,5	A/A	0,3
2	139632978	rs336320431	T	C	KLHL3	0.500	0.636	0.667	0.167	FALSE	1.000	0.363	0.192	0.016	T/C	7,4	T/C	6,6	T/C	10,2	T/C	5,10	T/T	3,0	T/T	7,0	C/C	0,7	C/C	0,3
2	139633098	rs332227027	G	A	KLHL3	0.500	0.667	0.200	0.778	FALSE	1.000	0.410	0.016	0.086	G/A	4,2	G/A	5,5	G/A	2,7	G/A	12,3	G/G	3,0	G/G	3,0	A/A	0,4	A/A	0,4
2	139633307	rs319242496	C	T	KLHL3	0.529	1.000	0.583	0.500	FALSE	0.808	0.004	0.563	1.000	C/C	6,0	C/T	9,8	C/T	8,8	C/T	5,7	C/C	5,0	C/C	3,0	T/T	0,9	T/T	0,4
2	148859693	rs335859347	C	T	JAKMIP2	0.364	0.625	0.667	0.667	FALSE	0.363	0.477	0.313	0.313	C/T	3,5	C/T	7,4	C/T	6,3	C/T	6,3	T/T	0,3	T/T	0,3	C/C	7,0	C/C	4,0
2	150518460	rs336456055	T	C	GRPEL2	0.350	0.625	0.500	0.615	FALSE	0.176	0.315	1.000	0.403	T/C	10,6	T/C	7,13	T/C	5,8	T/C	2,2	T/T	6,0	T/T	6,0	C/C	0,3	C/C	0,4
3	305199	rs325463824	A	G	PDGFA	0.696	0.773	0.375	0.484	FALSE	0.057	0.009	0.218	0.857	A/G	17,5	A/G	16,7	A/G	16,15	A/G	15,9	A/A	5,0	A/A	5,0	G/G	0,4	G/G	0,4
3	1861766	rs332729117	C	G	IQCE	0.600	0.714	0.684	0.706	FALSE	0.437	0.249	0.104	0.085	C/G	2,5	C/G	6,9	C/G	12,5	C/G	13,6	G/G	0,4	G/G	0,3	C/C	3,0	C/C	3,0
3	6471984	rs319209392	C	T	ZNF789	1.000	0.500	0.000	0.000	FALSE	0.019	1.000	0.008	0.004	C/T	2,2	C/C	4,0	C/C	6,0	C/C	5,0	C/C	3,0	C/C	5,0	T/T	0,4	T/T	0,3
3	8022568	.	A	G	TAF6	0.111	0.500	0.000	0.429	FALSE	0.013	1.000	0.041	0.705	A/G	4,4	A/G	1,8	A/G	4,3	A/A	3,0	A/A	3,0	A/A	4,0	G/G	0,4	G/G	0,3
3	8560671	rs330092376	A	G	TFR2	0.222	0.000	0.900	0.727	FALSE	0.086	0.019	0.007	0.125	G/G	0,4	A/G	2,7	A/G	3,8	A/G	1,9	A/A	3,0	A/A	3,0	G/G	0,5	G/G	0,4
3	11347349	rs338519548	T	C	EIF4H	0.506	0.433	0.611	0.515	FALSE	0.910	0.301	0.181	0.808	T/C	34,26	T/C	39,40	T/C	35,33	T/C	22,14	C/C	0,4	C/C	0,3	T/T	4,0	T/T	7,0
3	11470070	rs337139458	G	A	ENSSSCG00000007719	0.316	0.375	0.429	0.500	FALSE	0.104	0.218	0.705	1.000	G/A	15,9	G/A	13,6	G/A	9,9	G/A	3,4	A/A	0,3	A/A	0,6	G/G	4,0	G/G	3,0
3	17362793	rs55618509	T	C	KAT8	0.957	0.500	0.533	0.600	FALSE	0.000	1.000	0.715	0.316	T/C	6,6	C/C	1,22	T/C	15,10	T/C	16,14	C/C	0,8	C/C	0,5	T/T	3,0	T/T	5,0
3	17955526	rs323918478	A	G	TBC1D10B	0.593	0.429	0.351	0.000	FALSE	0.335	0.449	0.068	0.000	A/G	16,12	A/G	11,16	G/G	0,35	A/G	13,24	G/G	0,5	G/G	0,5	A/A	6,0	A/A	5,0
3	17955528	rs334275749	T	C	TBC1D10B	0.593	0.429	0.351	0.000	FALSE	0.335	0.449	0.068	0.000	T/C	16,12	T/C	11,16	C/C	0,35	T/C	13,24	C/C	0,5	C/C	0,5	T/T	6,0	T/T	6,0
3	18542619	rs333562574	C	T	SH2B1	0.565	0.412	0.654	0.346	FALSE	0.531	0.466	0.114	0.114	C/T	7,10	C/T	13,10	C/T	17,9	C/T	9,17	C/C	4,0	C/C	5,0	T/T	0,3	T/T	1,2
3	18871014	rs327779043	G	A	XPO6	0.700	0.464	1.000	0.600	FALSE	0.070	0.705	0.041	0.526	G/A	15,13	G/A	6,14	G/A	6,4	G/G	3,0	A/A	0,7	A/A	0,5	G/G	5,0	G/G	4,0
3	18872381	rs322490278	T	C	XPO6	0.500	0.417	1.000	1.000	FALSE	1.000	0.413	0.004	0.000	T/C	14,10	T/C	8,8	T/T	26,0	T/T	6,0	C/C	0,3	C/C	0,5	T/T	3,0	T/T	4,0
3	18872384	rs331329101	T	C	XPO6	0.813	0.565	0.333	0.692	FALSE	0.009	0.531	0.410	0.047	T/C	10,13	T/C	3,13	T/C	18,8	T/C	2,4	C/C	0,3	C/C	0,5	T/T	3,0	T/T	4,0
3	26160466	rs328344069	T	C	CCP110	0.190	0.273	0.640	0.609	FALSE	0.003	0.125	0.159	0.295	T/C	3,8	T/C	4,17	T/C	9,14	T/C	9,16	T/T	4,0	T/T	3,0	C/C	0,3	C/C	0,8
3	26196433	rs81216812	C	T	GDE1	0.000	0.129	0.643	0.421	FALSE	0.000	0.000	0.282	0.490	C/T	4,27	T/T	0,30	C/T	11,8	C/T	5,9	C/C	3,0	C/C	8,0	T/T	0,3	T/T	0,4
3	26769805	rs692747208	A	G	ENSSSCG00000025281	0.434	0.257	0.427	0.500	FALSE	0.251	0.000	0.203	1.000	A/G	55,19	A/G	43,33	A/G	42,42	A/G	32,43	G/G	0,3	G/G	0,3	A/A	7,0	A/A	6,0
3	31271649	rs699636182	A	G	GSPT1	1.000	0.200	0.000	1.000	FALSE	0.008	0.165	0.041	0.041	A/G	1,4	A/A	5,0	G/G	0,3	A/A	3,0	A/A	3,0	A/A	5,0	G/G	0,4	G/G	0,3
3	31272736	rs701621625	T	C	GSPT1	0.714	0.545	0.500	0.778	FALSE	0.046	0.763	1.000	0.086	T/C	6,5	T/C	15,6	T/C	2,7	T/C	2,2	T/T	4,0	T/T	3,0	C/C	0,3	C/C	0,7
3	32309899	rs336760914	C	T	TVP23A	0.714	0.538	0.600	0.667	FALSE	0.249	0.781	0.526	0.244	C/T	7,6	C/T	5,2	C/T	4,8	C/T	4,6	C/C	3,0	C/C	3,0	T/T	0,4	T/T	0,3
3	32313452	rs338588905	C	G	NUBP1	0.438	0.545	0.400	0.585	FALSE	0.479	0.601	0.316	0.273	C/G	18,15	C/G	14,18	C/G	17,24	C/G	15,10	C/C	4,0	C/C	6,0	G/G	0,5	G/G	0,5
3	33909272	rs334918344	C	T	ABAT	0.462	0.643	0.690	0.419	FALSE	0.695	0.128	0.012	0.285	C/T	18,10	C/T	12,14	C/T	25,18	C/T	13,29	C/C	4,0	C/C	3,0	T/T	0,4	T/T	0,3
3	33909813	rs81369039	C	T	ABAT	0.387	0.439	0.600	0.574	FALSE	0.207	0.434	0.272	0.306	C/T	18,23	C/T	12,19	C/T	20,27	C/T	12,18	C/C	6,0	C/C	4,0	T/T	0,5	T/T	0,5
3	37653194	rs340091261	C	T	ENSSSCG00000007935	0.286	0.333	0.600	0.000	FALSE	0.249	0.410	0.654	0.041	C/T	2,4	C/T	2,5	C/C	3,0	C/T	2,3	C/C	6,0	C/C	5,0	T/T	0,3	T/T	0,5
3	37999115	rs345972695	T	C	GLIS2	0.533	0.333	0.545	0.474	FALSE	0.796	0.244	0.763	0.819	T/C	8,4	T/C	7,8	T/C	9,10	T/C	6,5	C/C	0,5	C/C	0,4	T/T	3,0	T/T	5,0
3	46168455	rs708825751	C	T	MRPS5	0.200	0.250	0.000	0.500	FALSE	0.165	0.306	0.019	1.000	C/T	1,3	C/T	1,4	C/T	2,2	C/C	4,0	C/C	6,0	C/C	4,0	T/T	0,5	T/T	0,6
3	69524651	rs320321353	A	G	CCT7	0.377	0.440	0.378	0.465	FALSE	0.054	0.396	0.137	0.647	A/G	28,22	A/G	38,23	A/G	20,23	A/G	14,23	G/G	0,3	G/G	0,4	A/A	3,0	A/A	4,0
3	106080807	rs332988688	T	C	FAM98A	0.600	0.600	0.500	0.667	FALSE	0.654	0.654	1.000	0.410	T/C	3,2	T/C	3,2	T/C	2,4	T/C	3,3	T/T	4,0	T/T	3,0	C/C	0,6	C/C	0,4
3	112033214	rs81216958	G	T	EMILIN1	0.300	0.375	0.533	0.500	FALSE	0.200	0.477	0.796	1.000	G/T	5,3	G/T	7,3	G/T	8,8	G/T	8,7	T/T	0,3	T/T	0,7	G/G	4,0	G/G	5,0
3	113468292	rs340086574	T	C	ENSSSCG00000008576	0.375	0.556	0.000	0.500	FALSE	0.315	0.739	0.041	1.000	T/C	4,5	T/C	10,6	T/C	2,2	C/C	0,3	C/C	0,6	C/C	0,4	T/T	5,0	T/T	5,0
3	126410266	rs1112458821	T	C	CYS1	0.500	0.615	1.000	0.571	FALSE	1.000	0.403	0.019	0.705	T/C	5,8	T/C	6,6	T/C	4,3	T/T	4,0	C/C	0,3	C/C	0,3	T/T	8,0	T/T	5,0
3	126411171	rs1113234677	G	A	CYS1	0.409	0.375	0.545	0.857	FALSE	0.392	0.218	0.763	0.001	G/A	15,9	G/A	13,9	G/A	18,3	G/A	6,5	A/A	0,4	A/A	0,4	G/G	3,0	G/G	6,0
3	126411180	rs1110740522	C	G	CYS1	0.333	0.346	0.500	0.842	FALSE	0.099	0.114	1.000	0.002	C/G	17,9	C/G	16,8	C/G	16,3	C/G	6,6	G/G	0,4	G/G	0,5	C/C	5,0	C/C	6,0
3	126411596	rs1109864539	A	C	CYS1	1.000	0.889	0.478	0.450	FALSE	0.000	0.000	0.835	0.654	A/C	2,16	C/C	0,28	A/C	9,11	A/C	11,12	C/C	0,5	C/C	0,3	A/A	3,0	A/A	4,0
3	126419415	rs707575205	C	T	KLF11	0.636	0.833	1.000	0.571	FALSE	0.363	0.088	0.008	0.705	C/T	1,5	C/T	4,7	C/T	4,3	C/C	5,0	T/T	0,6	T/T	0,3	C/C	6,0	C/C	6,0
3	127364028	rs321813578	G	A	MBOAT2	0.333	0.500	0.200	0.333	FALSE	0.410	1.000	0.165	0.560	G/A	4,4	G/A	4,2	G/A	1,2	G/A	1,4	A/A	0,3	A/A	0,4	G/G	5,0	G/G	4,0
4	907296	rs320068358	A	G	FAM83H	1.000	1.000	0.000	0.000	TRUE	0.041	0.019	0.008	0.019	G/G	0,4	G/G	0,3	G/G	0,4	G/G	0,5	G/G	0,5	G/G	0,3	A/A	3,0	A/A	3,0
4	15195895	.	A	G	RNF139	0.444	0.333	0.333	0.000	FALSE	0.739	0.313	0.560	0.041	A/G	6,3	A/G	5,4	G/G	0,3	A/G	1,2	G/G	0,3	G/G	0,3	A/A	3,0	A/A	3,0
4	15308565	rs333408461	G	A	TMEM65	0.563	0.625	0.333	0.500	FALSE	0.617	0.477	0.560	1.000	G/A	3,5	G/A	7,9	G/A	3,3	G/A	1,2	A/A	0,4	A/A	0,4	G/G	6,0	G/G	3,0
4	15309630	rs342090275	T	A	TMEM65	0.583	0.500	0.000	0.600	FALSE	0.563	1.000	0.019	0.654	T/A	4,4	T/A	5,7	T/A	3,2	A/A	0,4	A/A	0,3	A/A	0,4	T/T	6,0	T/T	3,0
4	15309716	rs324159150	A	G	TMEM65	0.333	0.400	0.286	0.500	FALSE	0.192	0.526	0.249	1.000	A/G	6,4	A/G	10,5	A/G	10,10	A/G	2,5	G/G	0,5	G/G	0,5	A/A	4,0	A/A	4,0
4	15928749	rs328088585	A	C	FBXO32	0.667	0.556	0.600	0.250	FALSE	0.410	0.739	0.654	0.306	A/C	4,5	A/C	2,4	A/C	1,3	A/C	3,2	C/C	0,3	C/C	0,6	A/A	6,0	A/A	3,0
4	16125770	rs320461488	T	A	ZHX1	0.368	0.542	0.571	0.267	FALSE	0.249	0.683	0.512	0.065	T/A	11,13	T/A	12,7	T/A	4,11	T/A	12,9	A/A	0,3	A/A	0,3	T/T	6,0	T/T	4,0
4	20935305	rs80914143	A	G	EXT1	0.517	0.739	0.515	0.448	FALSE	0.853	0.019	0.862	0.577	A/G	17,6	A/G	15,14	A/G	16,13	A/G	16,17	A/A	3,0	A/A	5,0	G/G	0,4	G/G	0,3
4	54930026	rs695297607	G	A	ZFAND1	0.300	0.500	0.333	1.000	FALSE	0.200	1.000	0.560	0.019	G/A	3,3	G/A	7,3	G/G	4,0	G/A	1,2	A/A	0,3	A/A	0,4	G/G	4,0	G/G	3,0
4	56602772	rs690965223	T	C	ENSSSCG00000006156	0.462	0.500	0.444	0.600	FALSE	0.781	1.000	0.739	0.526	T/C	2,2	T/C	7,6	T/C	6,4	T/C	4,5	C/C	0,3	C/C	0,3	T/T	5,0	T/T	6,0
4	61577533	rs320921555	C	T	GDAP1	0.563	0.333	0.588	0.577	FALSE	0.617	0.153	0.302	0.432	C/T	12,6	C/T	7,9	C/T	15,11	C/T	20,14	T/T	0,5	T/T	0,3	C/C	3,0	C/C	7,0
4	67751832	rs80855249	C	T	COPS5	0.618	0.448	0.333	0.455	FALSE	0.168	0.577	0.123	0.601	C/T	13,16	C/T	21,13	C/T	18,15	C/T	14,7	C/C	4,0	C/C	6,0	T/T	0,4	T/T	0,3
4	68485754	rs345608770	C	G	CRH	0.500	0.500	0.600	0.667	FALSE	1.000	1.000	0.437	0.192	C/G	8,8	C/G	6,6	C/G	5,10	C/G	6,9	C/C	4,0	C/C	3,0	G/G	0,4	G/G	0,6
4	68485766	rs332371738	T	A	CRH	0.500	0.500	0.600	0.647	FALSE	1.000	1.000	0.437	0.222	T/A	8,8	T/A	5,5	T/A	6,11	T/A	6,9	T/T	3,0	T/T	3,0	A/A	0,4	A/A	0,4
4	70980530	rs326785616	G	A	GGH	1.000	0.500	1.000	0.000	FALSE	0.019	1.000	0.041	0.004	G/A	2,2	A/A	0,4	A/A	0,6	G/G	3,0	A/A	0,4	A/A	0,5	G/G	3,0	G/G	3,0
4	78161674	rs340488443	G	A	PCMTD1	0.318	0.471	1.000	0.737	FALSE	0.084	0.808	0.008	0.035	G/A	9,8	G/A	15,7	G/A	5,14	G/A	0,5	G/G	3,0	A/A	0,4	G/G	3,0	A/A	0,4
4	78162034	rs323166631	G	A	PCMTD1	0.633	0.500	0.636	0.500	FALSE	0.142	1.000	0.363	1.000	G/A	7,7	G/A	11,19	G/A	10,10	G/A	4,7	G/G	4,0	G/G	0,5	G/G	3,0	A/A	0,4
4	95983134	rs55618313	C	T	ILF2	0.550	0.450	0.571	0.344	FALSE	0.527	0.527	0.592	0.075	C/T	22,18	C/T	18,22	C/T	11,21	C/T	8,6	T/T	0,5	T/T	0,3	C/C	6,0	C/C	6,0
4	97534018	rs321122293	G	A	SNX27	0.357	0.438	0.444	0.136	FALSE	0.282	0.617	0.563	0.000	G/A	9,7	G/A	9,5	G/A	3,19	G/A	12,15	A/A	0,3	A/A	0,7	G/G	3,0	G/G	4,0
4	97994394	rs80932186	G	A	ENSSSCG00000034144	0.667	0.222	0.000	0.000	FALSE	0.410	0.086	0.019	0.000	G/A	2,7	G/A	4,2	G/G	14,0	G/G	4,0	G/G	3,0	A/A	0,8	A/A	3,0	A/A	0,3
4	99381671	rs342368881	G	A	ANKRD34A	0.440	0.429	0.500	0.621	FALSE	0.396	0.354	1.000	0.191	G/A	24,18	G/A	28,22	G/A	18,11	G/A	15,15	A/A	0,4	A/A	0,3	G/G	3,0	G/G	6,0
4	99947129	rs80956733	T	C	ACP6	0.619	0.313	0.231	0.148	FALSE	0.273	0.129	0.005	0.000	T/C	11,5	T/C	8,13	T/C	4,23	T/C	6,20	C/C	0,4	C/C	0,4	T/T	4,0	T/T	3,0
4	109921555	rs1113046377	A	G	SLC6A17	0.897	0.667	0.094	0.329	TRUE	0.000	0.002	0.000	0.002	A/G	27,54	A/G	9,78	A/G	26,53	A/G	6,58	G/G	0,3	G/G	0,3	A/A	4,0	A/A	5,0
4	110991411	rs80784587	C	T	TAF13	0.696	0.821	0.524	0.433	FALSE	0.057	0.000	0.827	0.465	C/T	5,23	C/T	7,16	C/T	13,17	C/T	11,10	T/T	0,6	T/T	0,4	C/C	3,0	C/C	3,0

4	117921625	rs330220593	A	T	DBT	0.538	0.429	0.200	0.462	FALSE	0.781	0.705	0.165	0.781	A/T	4,3	A/T	6,7	A/T	6,7	A/T	1,4	T/T	0,3	T/T	0,4	A/A	7,0	A/A	7,0
4	118187130	rs330906837	G	A	AGL	0.200	0.000	1.000	0.667	FALSE	0.050	0.019	0.041	0.410	G/G	4,0	G/A	8,2	G/A	4,2	G/G	3,0	A/A	0,3	A/A	0,3	G/G	4,0	G/G	4,0
4	123389356	rs337458754	A	T	GCLM	0.625	0.600	1.000	1.000	FALSE	0.477	0.654	0.008	0.004	A/T	3,2	A/T	5,3	T/T	0,6	T/T	0,5	A/A	3,0	A/A	5,0	T/T	0,3	T/T	0,3
4	123389368	rs319312287	T	G	GCLM	0.500	0.667	1.000	1.000	FALSE	1.000	0.410	0.004	0.008	T/G	2,4	T/G	6,6	T/T	5,0	T/T	6,0	G/G	0,3	G/G	0,5	T/T	3,0	T/T	3,0
4	123389507	rs330751611	A	G	GCLM	0.286	0.444	1.000	1.000	FALSE	0.249	0.739	0.019	0.004	A/G	4,5	A/G	2,5	G/G	0,6	G/G	0,4	A/A	3,0	A/A	3,0	G/G	0,4	G/G	0,6
4	123389755	rs322400622	T	C	GCLM	0.414	0.462	1.000	0.833	FALSE	0.352	0.695	0.000	0.000	T/C	12,14	T/C	12,17	T/C	7,35	C/C	0,35	T/T	4,0	T/T	3,0	C/C	0,3	C/C	0,4
4	123408293	rs321447722	G	A	DNTTIP2	0.625	0.438	0.857	0.385	FALSE	0.477	0.617	0.005	0.403	G/A	7,9	G/A	5,3	G/A	8,5	G/A	2,12	G/G	5,0	G/G	4,0	A/A	0,3	A/A	0,3
4	123408730	rs340578862	T	C	DNTTIP2	0.423	0.471	0.833	0.750	FALSE	0.432	0.808	0.016	0.041	T/C	8,9	T/C	11,15	T/C	4,12	T/C	2,10	T/T	5,0	T/T	5,0	C/C	0,6	C/C	0,5
4	123408935	rs343068549	C	T	DNTTIP2	0.300	0.533	0.563	0.500	FALSE	0.200	0.796	0.617	1.000	C/T	8,7	C/T	3,7	C/T	6,6	C/T	7,9	C/C	3,0	C/C	7,0	T/T	0,5	T/T	0,4
4	123410060	rs702324439	T	C	DNTTIP2	0.500	0.571	0.667	1.000	FALSE	1.000	0.705	0.410	0.008	T/C	3,4	T/C	3,3	T/T	5,0	T/C	4,2	C/C	0,3	C/C	0,3	T/T	5,0	T/T	10,0
4	125310034	rs323200496	C	T	TGFBR3	0.333	1.000	0.333	0.000	FALSE	0.410	0.002	0.410	0.000	C/C	7,0	C/T	2,4	C/C	16,0	C/T	4,2	C/C	4,0	C/C	3,0	T/T	0,3	T/T	0,3
4	127507581	rs334188364	A	G	KYAT3	0.000	0.182	0.350	0.786	FALSE	0.000	0.028	0.176	0.002	A/G	9,2	A/A	18,0	A/G	22,6	A/G	7,13	G/G	0,4	G/G	0,3	A/A	5,0	A/A	3,0
4	129670916	rs319840267	C	T	CLCA2	1.000	0.667	1.000	0.571	FALSE	0.008	0.410	0.041	0.705	C/T	2,4	T/T	0,5	C/T	4,3	C/C	3,0	T/T	0,4	T/T	0,3	C/C	3,0	C/C	6,0
5	9615643	rs336164574	T	C	ENSSSCG00000000107	0.778	0.200	0.556	0.625	FALSE	0.086	0.165	0.739	0.477	T/C	1,4	T/C	7,2	T/C	5,3	T/C	5,4	C/C	0,3	T/T	3,0	C/C	0,4	T/T	3,0
5	11224897	rs81385897	C	T	EIF3D	0.674	0.714	0.578	0.420	FALSE	0.017	0.021	0.296	0.257	C/T	20,8	C/T	31,15	C/T	21,29	C/T	26,19	T/T	0,5	C/C	3,0	T/T	0,4	C/C	4,0
5	12668289	rs331727847	C	T	PRDM4	0.556	0.250	0.000	0.286	FALSE	0.739	0.148	0.001	0.249	C/T	2,6	C/T	5,4	C/T	5,2	C/C	8,0	C/C	3,0	C/C	3,0	T/T	0,5	T/T	0,5
5	16919711	rs325871415	C	A	SLC4A8	0.368	0.000	0.667	0.000	FALSE	0.249	0.001	0.560	0.019	A/A	4,0	C/A	7,12	C/C	4,0	C/A	1,2	C/C	5,0	C/C	3,0	A/A	0,3	A/A	0,5
5	16920526	rs339933602	A	G	SLC4A8	0.533	0.375	0.417	0.450	FALSE	0.796	0.315	0.563	0.654	A/G	6,10	A/G	8,7	A/G	11,9	A/G	7,5	A/A	3,0	A/A	4,0	G/G	0,6	G/G	0,4
5	22501427	rs338236419	C	T	LRP1	0.314	0.471	1.000	1.000	FALSE	0.026	0.808	0.041	0.019	C/T	9,8	C/T	24,11	C/C	4,0	C/C	3,0	T/T	0,4	T/T	0,3	C/C	5,0	C/C	4,0
5	23025450	.	G	A	AGAP2	0.571	0.617	0.435	0.476	FALSE	0.209	0.034	0.531	0.758	G/A	31,50	G/A	33,44	G/A	20,22	G/A	10,13	A/A	0,7	A/A	0,3	G/G	8,0	G/G	5,0
5	28275911	rs80948726	A	G	RXYLT1	0.583	0.222	0.400	0.417	FALSE	0.563	0.086	0.526	0.563	A/G	7,2	A/G	5,7	A/G	5,7	A/G	4,6	G/G	0,3	G/G	0,4	A/A	6,0	A/A	4,0
5	28275916	rs80827137	A	G	RXYLT1	0.545	0.222	0.400	0.455	FALSE	0.763	0.086	0.526	0.763	A/G	7,2	A/G	5,6	A/G	5,6	A/G	4,6	G/G	0,3	G/G	0,4	A/A	6,0	A/A	4,0
5	28603031	rs344252087	C	T	SRGAP1	0.500	0.286	0.000	0.667	FALSE	1.000	0.249	0.041	0.560	C/T	2,5	C/T	6,6	C/T	1,2	C/C	3,0	C/C	6,0	C/C	5,0	T/T	0,6	T/T	0,3
5	28603825	rs324272300	G	A	SRGAP1	0.467	0.500	0.333	0.455	FALSE	0.796	1.000	0.560	0.763	G/A	6,6	G/A	8,7	G/A	5,6	G/A	1,2	A/A	0,9	A/A	0,3	G/G	8,0	G/G	6,0
5	29297273	rs331970524	A	G	TBC1D30	0.333	0.714	0.750	0.500	FALSE	0.410	0.249	0.306	1.000	A/G	5,2	A/G	2,4	A/G	3,3	A/G	1,3	A/A	5,0	A/A	4,0	G/G	0,3	G/G	0,3
5	30698609	rs342221788	G	C	GRIP1	0.500	0.727	0.571	1.000	FALSE	1.000	0.125	0.705	0.019	G/C	3,8	G/C	4,4	G/G	4,0	G/C	4,3	C/C	0,4	C/C	0,4	G/G	8,0	G/G	3,0
5	37979611	.	G	A	ATXN7L3B	0.475	0.551	0.500	0.400	FALSE	0.696	0.475	1.000	0.526	G/A	22,27	G/A	31,28	G/A	4,6	G/A	2,2	A/A	0,3	A/A	0,5	G/G	6,0	G/G	5,0
5	39328492	rs328626033	A	G	ENSSSCG00000000522	0.609	0.450	0.400	0.538	FALSE	0.295	0.654	0.369	0.695	A/G	11,9	A/G	9,14	A/G	14,12	A/G	8,12	G/G	0,7	G/G	0,3	A/A	6,0	A/A	3,0
5	46032153	rs322364919	C	A	PPFIBP1	0.286	0.235	0.000	0.300	FALSE	0.103	0.025	0.019	0.070	C/A	13,4	C/A	10,4	C/A	6,14	A/A	0,4	A/A	0,4	A/A	0,7	C/C	4,0	C/C	3,0
5	46491458	rs327641968	G	A	STK38L	0.667	0.500	1.000	0.143	FALSE	0.410	1.000	0.041	0.047	G/A	2,2	G/A	2,4	G/A	1,6	G/G	3,0	A/A	0,9	A/A	0,3	G/G	6,0	G/G	6,0
5	57021957	rs324092941	G	A	PTPRO	0.429	0.143	0.600	0.500	FALSE	0.592	0.047	0.369	1.000	G/A	1,6	G/A	6,8	G/A	8,8	G/A	8,12	G/G	5,0	G/G	5,0	A/A	0,4	A/A	0,5
5	58023525	rs318767453	G	A	ATF7IP	0.636	0.400	0.333	0.700	FALSE	0.363	0.654	0.560	0.200	G/A	3,2	G/A	4,7	G/A	7,3	G/A	1,2	A/A	0,5	A/A	0,7	G/G	4,0	G/G	5,0
5	59503494	rs81216086	T	C	HEBP1	0.263	0.526	0.600	0.556	FALSE	0.035	0.819	0.654	0.637	T/C	9,10	T/C	14,5	T/C	10,8	T/C	3,2	C/C	0,5	C/C	0,4	T/T	3,0	T/T	4,0
5	63087008	rs1107889472	G	A	FOXJ2	0.556	1.000	0.600	0.500	FALSE	0.637	0.001	0.437	1.000	G/G	8,0	G/A	10,8	G/A	9,9	G/A	6,9	G/G	3,0	G/G	3,0	A/A	0,4	A/A	0,3
5	76809680	rs329805779	C	T	SCAF11	0.000	0.000	0.556	0.667	FALSE	0.019	0.041	0.739	0.410	C/C	3,0	C/C	4,0	C/T	4,2	C/T	5,4	T/T	0,6	T/T	0,3	C/C	9,0	C/C	3,0
5	80474007	rs333357236	A	G	TDG	1.000	0.385	0.833	0.400	FALSE	0.008	0.403	0.088	0.437	A/G	5,8	A/A	5,0	A/G	9,6	A/G	1,5	A/A	5,0	A/A	4,0	G/G	0,4	G/G	0,6
6	1011138	.	T	C	MVD	0.824	0.857	0.563	0.517	FALSE	0.005	0.005	0.617	0.853	T/C	12,2	T/C	14,3	T/C	14,15	T/C	7,9	T/T	4,0	T/T	4,0	C/C	0,3	C/C	0,4
6	17522706	rs81258147	T	C	CYB5B	0.500	0.563	0.571	0.500	FALSE	1.000	0.617	0.705	1.000	T/C	7,9	T/C	9,9	T/C	2,2	T/C	4,3	C/C	0,3	C/C	0,3	T/T	6,0	T/T	4,0
6	17522723	rs81258148	C	T	CYB5B	0.474	0.654	0.571	0.200	FALSE	0.819	0.114	0.705	0.165	C/T	9,17	C/T	10,9	C/T	1,4	C/T	4,3	T/T	0,3	T/T	0,3	C/C	7,0	C/C	4,0
6	17649361	rs81216513	C	T	ENSSSCG00000002759	0.786	0.750	0.500	0.571	FALSE	0.027	0.148	1.000	0.592	C/T	2,6	C/T	3,11	C/T	8,6	C/T	3,3	T/T	0,3	T/T	0,6	C/C	4,0	C/C	5,0
6	27511606	rs692186771	G	A	NAE1	0.667	0.500	0.545	0.778	FALSE	0.313	1.000	0.763	0.086	G/A	5,5	G/A	6,3	G/A	2,7	G/A	5,6	G/G	6,0	G/G	3,0	A/A	0,3	A/A	0,4
6	37605974	rs334242599	G	A	DNAJA2	0.500	0.375	0.600	0.556	FALSE	1.000	0.477	0.369	0.637	G/A	5,3	G/A	3,3	G/A	10,8	G/A	12,8	A/A	0,9	A/A	0,4	G/G	4,0	G/G	4,0
6	54234153	rs328398294	T	C	GYS1	0.000	0.500	0.200	0.333	FALSE	0.041	1.000	0.165	0.560	T/C	2,2	T/T	3,0	T/C	1,2	T/C	1,4	C/C	0,5	C/C	0,7	T/T	3,0	T/T	6,0
6	57934476	rs55618457	A	G	PPP2R1A	0.495	0.540	0.557	0.487	FALSE	0.924	0.423	0.286	0.780	A/G	46,54	A/G	56,55	A/G	56,59	A/G	49,39	G/G	0,4	G/G	0,3	A/A	5,0	A/A	3,0
6	68527666	rs697953301	G	T	PER3	0.400	0.000	0.333	0.400	FALSE	0.654	0.047	0.410	0.654	T/T	0,6	G/T	2,3	G/T	3,2	G/T	4,2	G/G	4,0	G/G	3,0	T/T	0,7	T/T	0,3
6	71915066	rs326584942	A	G	CLCN6	0.500	0.294	0.923	0.421	FALSE	1.000	0.085	0.001	0.490	A/G	5,12	A/G	5,5	A/G	11,8	G/G	1,12	A/A	7,0	A/A	3,0	G/G	0,5	G/G	0,3
6	78885623	rs81212896	G	A	DDOST	0.469	0.625	0.375	0.415	FALSE	0.724	0.218	0.112	0.273	G/A	15,9	G/A	15,17	G/A	24,17	G/A	25,15	G/G	3,0	G/G	3,0	A/A	0,3	A/A	0,3
6	87941401	rs319661007	A	G	ZCCHC17	0.587	0.513	0.500	0.415	FALSE	0.237	0.873	1.000	0.215	A/G	19,20	A/G	19,27	A/G	22,31	A/G	23,23	G/G	0,3	G/G	0,6	A/A	5,0	A/A	4,0
6	88260691	rs81389500	G	A	ADGRB2	0.643	0.273	0.857	0.846	FALSE	0.282	0.125	0.047	0.009	G/A	3,8	G/A	9,5	G/A	2,11	G/A	1,6	G/G	4,0	G/G	3,0	A/A	0,3	A/A	0,3
6	148940860	rs328266727	A	G	EFCAB7	0.556	1.000	0.200	0.000	FALSE	0.739	0.041	0.165	0.008	G/G	0,3	A/G	4,5	G/G	0,5	A/G	1,4	G/G	0,3	G/G	0,6	A/A	4,0	A/A	3,0
6	165701447	.	G	A	IPP	0.545	1.000	0.500	1.000	FALSE	0.763	0.001	1.000	0.041	G/G	8,0	G/A	6,5	A/A	0,3	G/A	2,2	G/G	4,0	G/G	4,0	A/A	0,3	A/A	0,8
6	165701841	.	A	G	IPP	0.636	0.600	0.583	0.357	FALSE	0.363	0.654	0.563	0.282	A/G	3,2	A/G	7,4	A/G	9,5	A/G	5,7	A/A	3,0	A/A	6,0	G/G	0,5	G/G	0,9
6	165701856	.	C	T	IPP	0.643	0.714	0.533	0.333	FALSE	0.282	0.249	0.796	0.244	C/T	5,2	C/T	9,5	C/T	8,4	C/T	7,8	C/C	4,0	C/C	5,0	T/T	0,5	T/T	0,8
6	165705409	.	G	T	TMEM69	1.000	0.667	0.000	0.375	FALSE	0.001	0.410	0.019	0.477	G/T	4,2	G/G	8,0	G/T	5,3	G/G	4,0	G/G	3,0	G/G	4,0	T/T	0,6	T/T	0,4
6	165777247	rs81217624	G	A	NASP	0.633	0.364	0.474	0.545	FALSE	0.142	0.198	0.819	0.670	G/A	8,14	G/A	19,11	G/A	10,12	G/A	10,9	G/G	4,0	G/G	7,0	A/A	0,4	A/A	0,5
7	4935459	rs318959134	G	A	DSP	0.600	0.600	0.000	0.273	FALSE	0.526	0.654	0.001	0.125	G/A	3,2	G/A	6,4	G/A	8,3	G/G	8,0	G/G	5,0	G/G	6,0	A/A	0,8	A/A	0,3
7	40911686	rs324731881	G	A	ENPP4	0.500	0.500	1.000	0.000	FALSE	1.000	1.000	0.019	0.041	G/A	3,3	G/A	5,5	G/G	3,0	A/A	0,4	G/G	3,0	G/G	3,0	A/A	0,5	A/A	0,6
7	40913402	rs321556705	G	A	ENPP4	0.500	0.200	0.714	0.545	FALSE	1.000	0.165	0.249	0.763	G/A	1,4	G/A	2,2	G/A	5,6	G/A	2,5	G/G	4,0	G/G	5,0	A/A	0,5	A/A	0,3
7	40920144	rs332261036	C	T	ENPP5	0.769	0.867	0.190	0.412	FALSE	0.046	0.003	0.003	0.466	C/T	13,2	C/T	10,3	C/T	10,7	C/T	17,4	C/C	6,0	C/C	4,0	T/T	0,8	T/T	0,5
7	40920426	rs331562342	G	A	ENPP5	0.609	0.500	0.500	0.500	FALSE	0.295	1.000	1.000	1.000	G/A	11,11	G/A	14,9	G/A	14,14	G/A	12,12	G/G	3,0	G/G	6,0	A/A	0,5	A/A	0,6
7	41453619	rs343118058	G	A	SLC25A27	0.000	0.105	0.444	0.667	FALSE	0.000	0.000	0.739	0.313	G/A	2,17	A/A	0,27	G/A	3,6	G/A	5,4	G/G	8,0	G/G	3,0	A/A	0,6	A/A	0,5
7	41454217	rs331667090	G	A	SLC25A27	0.000	0.111	0.375	0.286	FALSE	0.000	0.000	0.477	0.103	G/A	3,24	A/A	0,24	G/A	10,4	G/A	5,3	G/G	4,0	G/G	4,0	A/A	0,3	A/A	0,6
7	41454237	rs339420250	A	G	SLC25A27	0.000	0.154	0.286	0.333	FALSE	0.000	0.000	0.249	0.153	A/G	4,22	G/G	0,25	A/G	12,6	A/G	5,2	A/A	4,0	A/A	4,0	G/G	0,3	G/G	0,5
7	41456661	.	G	A	SLC25A27	0.667	0.571	0.600	0.000	FALSE	0.313	0.705	0.654	0.041	G/A	4,3	G/A	6,3	G/G	3,0	G/A	2,3	G/G	3,0	G/G	7,0	A/A	0,3	A/A	0,7
7	46232691	rs1110027088	G	A	PAQR8	0.545	0.000	0.333	0.714	FALSE	0.763	0.041	0.560	0.249	G/G	3,0	G/A	5,6	G/A	5,2	G/A	1,2	A/A	0,6	A/A	0,6	G/G	3,0	G/G	4,0
7	49447055	rs339653126	G	A	ARNT2	0.000	0.244	0.333	0.500	FALSE	0.000	0.001	0.313	1.000	G/A	10,31	A/A	0,45	G/A	14,14	G/A	6,3	G/G	4,0	G/G	5,0	A/A	0,6	A/A	0,5

7	64582886	rs80865589	A	G	PSMA6	0.000	0.194	0.525	0.529	FALSE	0.000	0.000	0.752	0.674	A/G	29,7	A/A	44,0	A/G	27,24	A/G	21,19	G/G	0,5	G/G	0,6	A/A	4,0	A/A	4,0
7	66922530	rs327738337	A	G	AKAP6	0.000	0.286	0.364	0.455	FALSE	0.000	0.021	0.363	0.763	A/G	20,8	A/A	26,0	A/G	5,6	A/G	4,7	G/G	0,7	G/G	0,3	A/A	6,0	A/A	4,0
7	103096967	rs341304760	C	T	DIO2	0.606	0.536	0.517	0.468	FALSE	0.221	0.705	0.853	0.662	C/T	15,13	C/T	20,13	C/T	25,22	C/T	14,15	C/C	3,0	C/C	5,0	T/T	0,5	T/T	0,7
7	110783872	rs336882201	T	C	TTC8	1.000	1.000	0.714	0.500	FALSE	0.004	0.041	0.249	1.000	T/T	3,0	T/T	6,0	T/C	3,3	T/C	5,2	C/C	0,5	T/T	4,0	C/C	0,5	T/T	3,0
7	114248806	rs340571749	T	C	GOLGA5	0.600	0.286	0.500	0.000	FALSE	0.654	0.249	1.000	0.008	T/C	2,5	T/C	3,2	T/T	5,0	T/C	2,2	T/T	3,0	T/T	3,0	C/C	0,3	C/C	0,6
7	114261992	rs80989621	A	C	GOLGA5	0.333	0.545	0.000	0.400	FALSE	0.313	0.763	0.008	0.526	A/C	6,5	A/C	3,6	A/C	6,4	A/A	5,0	A/A	6,0	A/A	4,0	C/C	0,9	C/C	0,3
8	3208109	rs338644446	G	C	SORCS2	1.000	1.000	0.818	0.667	FALSE	0.002	0.000	0.028	0.313	C/C	0,9	C/C	0,7	G/C	3,6	G/C	2,9	G/G	7,0	C/C	0,4	G/G	4,0	C/C	0,6
8	10868171	rs326120215	A	G	CC2D2A	0.571	0.667	0.500	0.600	FALSE	0.705	0.560	1.000	0.654	A/G	1,2	A/G	3,4	A/G	3,2	A/G	2,2	G/G	0,4	G/G	0,4	A/A	6,0	A/A	4,0
8	11367298	rs330706159	G	A	TAPT1	0.600	0.429	0.750	0.667	FALSE	0.654	0.705	0.306	0.313	G/A	3,4	G/A	3,2	G/A	3,6	G/A	1,3	G/G	8,0	G/G	3,0	A/A	0,8	A/A	0,5
8	29102082	rs345302250	A	G	C4orf19	0.500	0.615	0.571	0.250	FALSE	1.000	0.403	0.705	0.148	A/G	5,8	A/G	3,3	A/G	2,6	A/G	4,3	G/G	0,3	G/G	0,4	A/A	4,0	A/A	5,0
8	29119565	rs340892322	T	C	RELL1	0.714	0.667	1.000	1.000	FALSE	0.249	0.560	0.019	0.008	T/C	1,2	T/C	2,5	T/T	5,0	T/T	4,0	C/C	0,5	C/C	0,3	T/T	3,0	T/T	7,0
8	36199678	rs319808296	T	C	GABRG1	0.500	0.348	0.667	0.500	FALSE	1.000	0.141	0.313	1.000	T/C	15,8	T/C	8,8	T/C	5,5	T/C	6,3	C/C	0,6	C/C	0,7	T/T	4,0	T/T	4,0
8	36876760	rs339376748	G	A	GABRA4	0.409	0.600	0.400	0.464	FALSE	0.392	0.369	0.369	0.705	G/A	8,12	G/A	13,9	G/A	13,15	G/A	8,12	A/A	0,4	A/A	0,5	G/G	5,0	G/G	4,0
8	39128341	rs325725228	G	A	SGCB	0.435	0.214	0.636	0.500	FALSE	0.531	0.002	0.363	1.000	G/A	22,6	G/A	13,10	G/A	7,7	G/A	7,4	A/A	0,5	A/A	0,4	G/G	7,0	G/G	4,0
8	39129214	rs333400626	C	T	SGCB	0.529	0.211	1.000	1.000	FALSE	0.808	0.009	0.041	0.004	C/T	15,4	C/T	8,9	C/C	6,0	C/C	3,0	T/T	0,7	T/T	0,3	C/C	8,0	C/C	5,0
8	39130557	rs338743264	C	T	SGCB	0.529	0.281	0.333	0.400	FALSE	0.808	0.012	0.560	0.654	C/T	23,9	C/T	8,9	C/T	2,3	C/T	1,2	T/T	0,7	T/T	0,5	C/C	4,0	C/C	5,0
8	41492774	rs319439230	C	T	KIT	0.231	0.200	0.529	0.600	FALSE	0.005	0.000	0.808	0.369	C/T	28,7	C/T	20,6	C/T	12,8	C/T	9,8	T/T	0,4	T/T	0,3	C/C	9,0	C/C	5,0
8	71792929	rs340775389	G	C	ENSSSCG00000008979	0.400	0.500	1.000	0.500	FALSE	0.654	1.000	0.041	1.000	G/C	2,2	G/C	2,3	G/C	2,2	G/G	3,0	C/C	0,5	G/G	4,0	C/C	0,3	G/G	5,0
8	75344764	rs326578340	C	A	SFRP2	0.500	0.417	0.500	0.636	FALSE	1.000	0.413	1.000	0.198	C/A	14,10	C/A	18,18	C/A	14,8	C/A	9,9	A/A	0,7	A/A	0,5	C/C	3,0	C/C	5,0
8	86516424	rs81402203	C	T	TBC1D9	0.600	0.433	0.625	0.556	FALSE	0.204	0.465	0.477	0.637	C/T	17,13	C/T	16,24	C/T	10,8	C/T	5,3	T/T	0,5	T/T	0,4	C/C	6,0	C/C	3,0
8	86517624	rs321578365	A	C	TBC1D9	0.455	0.667	0.368	0.367	FALSE	0.601	0.080	0.249	0.142	A/C	9,18	A/C	18,15	A/C	11,19	A/C	7,12	C/C	0,4	C/C	0,3	A/A	3,0	A/A	3,0
8	118153531	rs81214183	T	C	CISD2	0.476	0.538	0.333	0.471	FALSE	0.827	0.781	0.560	0.808	T/C	6,7	T/C	11,10	T/C	8,9	T/C	1,2	C/C	0,4	C/C	0,4	T/T	3,0	T/T	3,0
8	118361691	rs340787108	T	G	MANBA	0.000	0.000	1.000	1.000	TRUE	0.041	0.041	0.019	0.041	T/T	3,0	T/T	3,0	T/T	3,0	T/T	4,0	G/G	0,6	G/G	0,5	T/T	3,0	T/T	3,0
8	121886409	rs345607897	G	A	RAP1GDS1	0.600	0.841	0.583	0.435	FALSE	0.178	0.000	0.563	0.531	G/A	7,37	G/A	18,27	G/A	10,13	G/A	7,5	A/A	0,3	A/A	0,6	G/G	6,0	G/G	5,0
8	130346374	rs697352154	C	A	HERC3	0.273	0.000	0.000	0.750	FALSE	0.125	0.000	0.000	0.076	A/A	0,11	C/A	3,8	C/A	3,9	C/C	16,0	C/C	6,0	C/C	5,0	A/A	0,4	A/A	0,3
8	130346375	rs81294933	A	G	HERC3	0.200	0.000	0.000	0.750	FALSE	0.050	0.000	0.000	0.076	G/G	0,11	A/G	2,8	A/G	3,9	A/A	16,0	A/A	6,0	A/A	5,0	G/G	0,4	G/G	0,3
8	131416404	rs323291039	G	C	SPARCL1	0.066	0.228	0.621	0.420	FALSE	0.000	0.000	0.191	0.184	G/C	29,98	C/C	8,114	G/C	29,40	G/C	18,11	C/C	0,3	G/G	3,0	C/C	0,3	G/G	6,0
9	8317493	rs343015936	C	G	PAAF1	0.500	0.455	0.636	1.000	FALSE	1.000	0.763	0.363	0.000	C/G	6,5	C/G	5,5	C/C	9,0	C/G	7,4	G/G	0,4	G/G	0,3	C/C	5,0	C/C	5,0
9	17577127	rs341089144	A	T	DLG2	0.426	0.350	0.565	0.543	FALSE	0.306	0.056	0.531	0.555	A/T	26,14	A/T	27,20	A/T	25,21	A/T	13,10	T/T	0,6	T/T	0,11	A/A	8,0	A/A	5,0
9	23184785	rs339345778	A	G	CHORDC1	0.556	0.545	0.500	0.000	FALSE	0.637	0.763	1.000	0.008	A/G	6,5	A/G	10,8	A/A	5,0	A/G	4,4	A/A	4,0	A/A	5,0	G/G	0,4	G/G	0,4
9	23185140	rs692909573	T	A	CHORDC1	0.400	0.200	1.000	0.833	FALSE	0.526	0.165	0.041	0.016	T/A	1,4	T/A	4,6	T/A	2,10	A/A	0,3	T/T	3,0	T/T	3,0	A/A	0,6	A/A	0,4
9	41418053	rs335414745	G	A	USP28	0.556	0.333	0.357	0.500	FALSE	0.739	0.244	0.282	1.000	G/A	4,8	G/A	5,4	G/A	10,10	G/A	9,5	G/G	3,0	G/G	4,0	A/A	0,5	A/A	0,4
9	46565521	rs322817809	G	A	USP2	0.400	0.571	0.462	0.471	FALSE	0.316	0.592	0.781	0.808	G/A	8,6	G/A	10,15	G/A	9,8	G/A	7,6	G/G	4,0	G/G	3,0	A/A	0,4	A/A	0,5
9	58534391	rs340574949	A	C	ENSSSCG00000031293	0.455	0.000	0.143	0.353	FALSE	0.601	0.000	0.005	0.222	C/C	0,26	A/C	15,18	A/C	11,6	A/C	12,2	A/A	5,0	A/A	5,0	C/C	0,7	C/C	0,5
9	74346497	rs340387131	A	T	CASD1	0.483	0.462	0.727	0.429	FALSE	0.853	0.695	0.030	0.449	A/T	14,12	A/T	15,14	A/T	12,16	A/T	16,6	T/T	0,6	T/T	0,4	A/A	7,0	A/A	7,0
9	74485347	rs336355554	C	T	PEG10	1.000	1.000	1.000	1.000	TRUE	0.000	0.000	0.000	0.000	T/T	0,51	T/T	0,47	C/C	34,0	C/C	24,0	T/T	0,4	T/T	0,3	C/C	5,0	C/C	5,0
9	80642777	rs81218348	A	G	ENSSSCG00000024313	0.643	0.608	0.172	0.434	FALSE	0.001	0.014	0.000	0.104	A/G	51,79	A/G	50,90	A/G	66,86	A/G	22,106	G/G	0,3	G/G	0,3	A/A	3,0	A/A	6,0
9	81863264	rs326972061	G	T	TMEM106B	0.316	0.261	0.500	0.600	FALSE	0.104	0.019	1.000	0.526	G/T	17,6	G/T	13,6	G/T	6,4	G/T	5,5	T/T	0,7	T/T	0,8	G/G	11,0	G/G	3,0
9	81863455	rs338735952	T	C	TMEM106B	0.348	0.667	0.647	0.500	FALSE	0.141	0.099	0.222	1.000	T/C	8,16	T/C	15,8	T/C	11,11	T/C	11,6	C/C	0,3	C/C	0,5	T/T	5,0	T/T	5,0
9	81863648	rs340816812	C	T	TMEM106B	0.561	0.462	0.517	0.565	FALSE	0.434	0.631	0.853	0.376	C/T	21,18	C/T	18,23	C/T	26,20	C/T	15,14	T/T	0,6	T/T	0,8	C/C	7,0	C/C	3,0
9	83323339	rs318389968	A	G	ETV1	0.520	0.375	0.308	0.333	FALSE	0.841	0.477	0.160	0.244	A/G	5,3	A/G	12,13	A/G	4,8	A/G	4,9	G/G	0,6	G/G	0,6	A/A	4,0	A/A	4,0
9	83570427	rs324860735	G	A	DGKB	0.700	0.462	0.455	0.444	FALSE	0.200	0.781	0.763	0.739	G/A	7,6	G/A	3,7	G/A	4,5	G/A	5,6	A/A	0,6	A/A	0,4	G/G	6,0	G/G	3,0
9	83570729	rs322604763	T	G	DGKB	0.400	0.500	0.600	0.625	FALSE	0.526	1.000	0.654	0.315	T/G	9,9	T/G	6,4	T/G	10,6	T/G	3,2	G/G	0,3	G/G	0,5	T/T	4,0	T/T	6,0
9	83572475	rs318283893	A	G	DGKB	0.533	0.545	0.600	0.600	FALSE	0.796	0.763	0.654	0.654	A/G	5,6	A/G	7,8	A/G	3,2	A/G	3,2	G/G	0,3	G/G	0,5	A/A	5,0	A/A	3,0
9	83572546	rs338269056	T	G	DGKB	0.286	0.588	0.500	0.667	FALSE	0.103	0.466	1.000	0.244	T/G	7,10	T/G	10,4	T/G	8,4	T/G	3,3	G/G	0,6	G/G	0,4	T/T	7,0	T/T	7,0
9	85850950	rs339909758	G	C	ANKMY2	0.481	0.480	0.467	0.600	FALSE	0.847	0.841	0.796	0.437	G/C	13,12	G/C	14,13	G/C	9,6	G/C	7,8	C/C	0,4	C/C	0,3	G/G	5,0	G/G	3,0
9	85851573	rs336043449	C	A	ANKMY2	0.571	0.400	0.429	0.500	FALSE	0.512	0.369	0.705	1.000	C/A	12,8	C/A	9,12	C/A	5,5	C/A	3,4	A/A	0,3	A/A	0,3	C/C	3,0	C/C	3,0
9	88762083	.	A	T	TWISTNB	0.667	0.375	0.333	0.333	FALSE	0.560	0.477	0.560	0.313	A/T	5,3	A/T	1,2	A/T	3,6	A/T	1,2	T/T	0,4	T/T	0,3	A/A	3,0	A/A	3,0
9	90958986	rs318562183	C	A	RAPGEF5	0.600	0.464	0.481	0.448	FALSE	0.235	0.705	0.847	0.577	C/A	15,13	C/A	14,21	C/A	13,16	C/A	13,14	A/A	0,4	A/A	0,4	C/C	3,0	C/C	6,0
9	92299349	rs326266859	T	C	TRA2A	0.600	0.300	0.517	0.630	FALSE	0.369	0.070	0.853	0.175	T/C	14,6	T/C	8,12	T/C	17,10	T/C	15,14	C/C	0,6	C/C	0,5	T/T	7,0	T/T	4,0
9	93132894	rs338460618	C	T	ENSSSCG00000015390	0.400	0.412	0.250	0.500	FALSE	0.526	0.466	0.306	1.000	C/T	10,7	C/T	6,4	C/T	3,3	C/T	1,3	T/T	0,5	T/T	0,6	C/C	8,0	C/C	5,0
9	93713845	rs340254901	G	A	KIAA1324L	0.636	0.389	0.571	0.423	FALSE	0.198	0.344	0.592	0.432	G/A	11,7	G/A	8,14	G/A	11,15	G/A	8,6	A/A	0,3	A/A	0,3	G/G	4,0	G/G	5,0
9	107474144	rs329846589	C	A	CBLL1	0.400	0.429	0.600	0.250	FALSE	0.654	0.705	0.654	0.306	C/A	4,3	C/A	3,2	C/A	1,3	C/A	3,2	A/A	0,8	A/A	0,5	C/C	3,0	C/C	4,0
9	107668404	rs324517808	T	C	DLD	0.313	0.267	0.364	0.500	FALSE	0.129	0.065	0.363	1.000	T/C	11,4	T/C	11,5	T/C	7,7	T/C	4,7	C/C	0,3	C/C	0,3	T/T	7,0	T/T	8,0
9	107668572	rs342441550	A	G	DLD	0.300	0.000	0.444	0.750	FALSE	0.200	0.019	0.739	0.076	A/A	4,0	A/G	7,3	A/G	9,3	A/G	4,5	G/G	0,5	G/G	0,5	A/A	4,0	A/A	5,0
9	107671777	rs81216553	C	A	LAMB1	0.407	0.360	0.417	0.545	FALSE	0.335	0.159	0.413	0.601	C/A	16,9	C/A	16,11	C/A	18,15	C/A	10,14	A/A	0,4	A/A	0,9	C/C	5,0	C/C	5,0
9	108862484	rs346014582	A	G	AMPH	0.667	0.500	0.000	0.000	FALSE	0.043	1.000	0.041	0.008	A/G	18,18	A/G	24,12	A/A	5,0	A/A	3,0	A/A	7,0	A/A	6,0	G/G	0,5	G/G	0,4
9	108862499	rs81208959	A	G	AMPH	0.619	0.526	0.000	0.000	FALSE	0.121	0.746	0.019	0.008	A/G	20,18	A/G	26,16	A/A	5,0	A/A	4,0	A/A	7,0	A/A	6,0	G/G	0,4	G/G	0,4
9	109742635	rs319654358	A	G	ENSSSCG00000034739	0.947	0.857	0.000	0.600	FALSE	0.000	0.001	0.019	0.526	A/G	3,18	G/G	1,18	A/G	6,4	G/G	0,4	G/G	0,3	G/G	0,7	A/A	4,0	A/A	3,0
9	118651712	rs340855629	C	T	ASTN1	0.216	0.000	1.000	0.476	FALSE	0.000	0.000	0.004	0.827	C/C	40,0	C/T	29,8	C/T	10,11	C/C	6,0	T/T	0,3	T/T	0,4	C/C	4,0	C/C	5,0
9	124498698	rs324937774	A	G	NMNAT2	0.288	0.000	0.294	0.382	FALSE	0.001	0.000	0.003	0.078	A/A	37,0	A/G	42,17	A/G	34,21	A/G	36,15	A/A	4,0	G/G	0,5	A/A	3,0	G/G	0,3
9	133351610	rs319331240	A	G	CAMK1G	0.333	0.286	0.484	0.500	FALSE	0.153	0.103	0.857	1.000	A/G	10,4	A/G	12,6	A/G	21,21	A/G	15,16	G/G	0,4	G/G	0,4	A/A	6,0	A/A	3,0
9	133352328	rs1110583121	T	G	CAMK1G	0.414	0.389	0.667	0.667	FALSE	0.352	0.344	0.080	0.053	T/G	11,7	T/G	17,12	T/G	22,11	T/G	18,9	G/G	0,4	G/G	0,4	T/T	9,0	T/T	3,0
9	133352604	rs81418639	C	G	CAMK1G	0.550	0.150	0.467	0.500	FALSE	0.654	0.001	0.796	1.000	C/G	17,3	C/G	9,11	C/G	10,10	C/G	7,8	G/G	0,3	G/G	0,4	C/C	4,0	C/C	3,0
9	134937227	rs320871666	T	C	CD34	0.615	0.444	0.462	0.563	FALSE	0.403	0.739	0.781	0.617	T/C	5,4	T/C	5,8	T/C	9,7	T/C	6,7	C/C	0,3	C/C	0,6	T/T	4,0	T/T	5,0
9	135026279	rs327778327	C	T	ENSSSCG00000038506	0.550	0.500	0.444	0.789	FALSE	0.654	1.000	0.739	0.009	C/T	7,7	C/T	9,11	C/T	15,4	C/T	4,5	T/T	0,4	T/T	0,4	C/C	5,0	C/C	4,0

9	135026802	rs339445894	C	T	ENSSSCG00000038506	0.625	0.500	0.333	1.000	FALSE	0.477	1.000	0.560	0.002	C/T	5,5	C/T	3,5	C/C	7,0	C/T	1,2	T/T	0,3	T/T	0,5	C/C	5,0	C/C	6,0
9	136582890	rs335201757	A	G	GRB10	0.000	0.500	0.286	0.400	FALSE	0.041	1.000	0.249	0.526	A/G	2,2	A/A	3,0	A/G	4,6	A/G	2,5	G/G	0,4	G/G	0,3	A/A	6,0	A/A	6,0
10	761980	rs1110270459	C	T	UCHL5	0.727	0.423	0.200	0.000	FALSE	0.030	0.432	0.016	0.000	C/T	15,11	C/T	6,16	C/C	22,0	C/T	12,3	C/C	5,0	T/T	0,4	C/C	4,0	T/T	0,5
10	9599062	rs344324532	A	G	EPRS	0.769	0.875	0.600	0.667	FALSE	0.046	0.001	0.654	0.560	A/G	2,14	A/G	3,10	A/G	1,2	A/G	2,3	A/A	5,0	G/G	0,5	A/A	4,0	G/G	0,3
10	10104657	rs196959944	C	G	MARK1	0.667	0.432	0.529	0.660	FALSE	0.065	0.410	0.732	0.022	C/G	16,21	C/G	20,10	C/G	17,33	C/G	16,18	C/C	4,0	C/C	3,0	G/G	0,8	G/G	0,4
10	12411910	rs81215804	G	A	FH	0.538	0.458	0.650	0.595	FALSE	0.631	0.683	0.176	0.248	G/A	11,13	G/A	21,18	G/A	15,22	G/A	7,13	G/G	7,0	G/G	5,0	A/A	0,5	A/A	0,4
10	17415171	rs320568975	A	G	DESI2	0.500	1.000	0.333	0.500	FALSE	1.000	0.019	0.410	1.000	A/A	4,0	A/G	4,4	A/G	4,4	A/G	2,4	G/G	0,7	A/A	3,0	G/G	0,3	A/A	3,0
10	17415562	rs81221844	A	G	DESI2	0.571	0.500	0.000	0.333	FALSE	0.705	1.000	0.019	0.410	A/G	3,3	A/G	4,3	A/G	2,4	G/G	0,4	G/G	0,4	A/A	3,0	G/G	0,3	A/A	5,0
10	24453878	rs342018632	T	C	UBE2T	0.400	1.000	0.533	1.000	FALSE	0.526	0.004	0.796	0.000	T/T	6,0	T/C	4,6	C/C	0,12	T/C	7,8	T/T	3,0	T/T	4,0	C/C	0,4	C/C	0,3
10	32895865	rs319261750	T	C	UBE2R2	0.333	0.471	0.000	0.375	FALSE	0.080	0.808	0.008	0.477	T/C	8,9	T/C	9,18	T/C	5,3	T/T	5,0	T/T	3,0	T/T	3,0	C/C	0,4	C/C	0,7
10	43902035	rs344926808	T	C	HACD1	0.571	0.500	0.750	0.500	FALSE	0.705	1.000	0.306	1.000	T/C	6,6	T/C	4,3	T/C	4,4	T/C	1,3	T/T	3,0	T/T	4,0	C/C	0,5	C/C	0,9
10	43902477	rs321389963	G	C	HACD1	0.250	0.200	0.000	0.500	FALSE	0.306	0.165	0.041	1.000	G/C	1,4	G/C	1,3	G/C	2,2	G/G	3,0	G/G	3,0	G/G	4,0	C/C	0,4	C/C	0,10
10	64595786	.	C	T	PRKCQ	0.250	0.500	1.000	0.500	FALSE	0.306	1.000	0.041	1.000	C/T	2,2	C/T	1,3	C/T	2,2	T/T	0,3	C/C	6,0	C/C	3,0	T/T	0,6	T/T	0,3
11	3258214	.	T	C	NUP58	0.286	0.529	0.333	0.700	FALSE	0.021	0.808	0.560	0.200	T/C	8,9	T/C	20,8	T/C	7,3	T/C	1,2	C/C	0,3	C/C	0,5	T/T	4,0	T/T	4,0
11	12314437	rs345017819	T	A	SPART	0.417	0.417	0.000	0.000	FALSE	0.563	0.563	0.004	0.001	T/A	5,7	T/A	5,7	T/T	8,0	T/T	6,0	T/T	3,0	T/T	3,0	A/A	0,9	A/A	0,8
11	19181555	rs335447437	C	A	RCBTB2	0.632	0.417	0.125	0.333	FALSE	0.249	0.413	0.024	0.244	C/A	14,10	C/A	7,12	C/A	4,8	C/A	1,7	A/A	0,4	A/A	0,4	C/C	3,0	C/C	5,0
11	19670078	rs703603372	C	A	SUCLA2	0.518	0.457	0.385	0.500	FALSE	0.789	0.555	0.148	1.000	C/A	21,25	C/A	29,27	C/A	26,26	C/A	24,15	C/C	3,0	C/C	4,0	A/A	0,7	A/A	0,3
11	19670080	rs712659535	A	G	SUCLA2	0.518	0.444	0.395	0.491	FALSE	0.789	0.456	0.193	0.891	A/G	20,25	A/G	29,27	A/G	27,26	A/G	23,15	A/A	3,0	A/A	4,0	G/G	0,7	G/G	0,3
11	21851661	rs1109872089	G	A	COG3	0.600	0.818	0.000	0.250	FALSE	0.526	0.028	0.041	0.306	G/A	2,9	G/A	4,6	G/A	1,3	A/A	0,3	A/A	0,3	A/A	0,5	G/G	3,0	G/G	3,0
11	25529634	rs343369338	T	C	NAA16	0.571	0.400	0.333	1.000	FALSE	0.705	0.654	0.560	0.041	T/C	3,2	T/C	3,4	T/T	3,0	T/C	1,2	C/C	0,3	C/C	0,3	T/T	6,0	T/T	8,0
11	67455236	rs1110300840	G	A	FARP1	0.500	0.412	0.818	1.000	FALSE	1.000	0.466	0.028	0.000	G/A	7,10	G/A	9,9	A/A	0,14	G/A	2,9	G/G	5,0	G/G	3,0	A/A	0,4	A/A	0,4
11	71159009	rs690055289	A	G	ERCC5	0.417	0.714	1.000	0.000	FALSE	0.563	0.249	0.041	0.041	A/G	2,5	A/G	7,5	A/A	3,0	G/G	0,3	A/A	3,0	G/G	0,5	A/A	4,0	G/G	0,4
11	71167703	rs332270865	T	G	ERCC5	0.000	0.000	0.438	0.583	FALSE	0.000	0.000	0.617	0.563	T/T	12,0	T/T	14,0	T/G	7,5	T/G	7,9	G/G	0,5	G/G	0,3	T/T	5,0	T/T	6,0
11	78609813	rs330500899	A	G	LAMP1	0.357	0.462	0.200	0.357	FALSE	0.282	0.781	0.165	0.282	A/G	7,6	A/G	9,5	A/G	5,9	A/G	1,4	G/G	0,4	G/G	0,8	A/A	9,0	A/A	4,0
12	632931	rs318890725	A	G	NARF	0.591	0.412	0.421	0.385	FALSE	0.392	0.466	0.490	0.237	A/G	10,7	A/G	9,13	A/G	10,16	A/G	8,11	G/G	0,3	G/G	0,4	A/A	4,0	A/A	4,0
12	685767	rs323461573	G	A	OGFOD3	0.462	1.000	0.667	0.320	FALSE	0.781	0.001	0.192	0.069	G/G	8,0	G/A	6,7	G/A	17,8	G/A	5,10	G/G	4,0	G/G	4,0	A/A	0,7	A/A	0,3
12	794242	rs341396677	C	T	CSNK1D	0.692	0.636	0.480	0.478	FALSE	0.160	0.363	0.841	0.835	C/T	7,4	C/T	9,4	C/T	12,11	C/T	13,12	C/C	4,0	C/C	3,0	T/T	0,5	T/T	0,3
12	6463848	rs334957540	A	G	NAT9	0.571	0.000	0.643	0.462	FALSE	0.592	0.019	0.282	0.781	A/A	4,0	A/G	6,8	A/G	6,7	A/G	9,5	G/G	0,3	G/G	0,4	A/A	6,0	A/A	4,0
12	6470821	rs336295300	G	A	SLC9A3R1	0.455	0.545	0.308	0.318	FALSE	0.670	0.763	0.160	0.084	G/A	5,6	G/A	12,10	G/A	7,15	G/A	4,9	A/A	0,4	A/A	0,3	G/G	4,0	G/G	5,0
12	11750354	rs328916319	T	C	WIPI1	1.000	0.500	0.000	0.000	FALSE	0.041	1.000	0.008	0.019	T/C	2,2	C/C	0,3	T/T	4,0	T/T	5,0	T/T	3,0	C/C	0,3	T/T	4,0	C/C	0,3
12	14490794	rs696063982	A	C	SMURF2	0.750	0.600	0.000	0.571	FALSE	0.306	0.526	0.041	0.705	A/C	4,6	A/C	1,3	A/C	3,4	A/A	3,0	A/A	5,0	C/C	0,3	A/A	4,0	C/C	0,6
12	15185982	rs346100249	A	G	CCDC47	0.600	0.500	0.375	0.125	FALSE	0.526	1.000	0.477	0.024	A/G	5,5	A/G	6,4	A/G	1,7	A/G	3,5	G/G	0,4	A/A	4,0	G/G	0,3	A/A	5,0
12	15398297	rs327022190	A	G	ACE	0.231	0.500	0.300	0.375	FALSE	0.046	1.000	0.200	0.315	A/G	6,6	A/G	3,10	A/G	6,10	A/G	3,7	G/G	0,3	A/A	4,0	G/G	0,4	A/A	3,0
12	16059898	rs321608862	C	A	MRC2	1.000	0.333	0.769	0.636	FALSE	0.004	0.560	0.046	0.363	C/A	1,2	C/C	6,0	C/A	4,7	C/A	3,10	C/C	7,0	C/C	5,0	A/A	0,6	A/A	0,4
12	16880361	rs333696937	A	C	CDC27	0.333	0.200	0.000	0.000	FALSE	0.313	0.165	0.041	0.041	A/C	1,4	A/C	3,6	A/A	3,0	A/A	3,0	A/A	4,0	A/A	4,0	C/C	0,6	C/C	0,7
12	17099252	rs339959681	C	A	KANSL1	0.525	0.398	0.452	0.477	FALSE	0.615	0.043	0.537	0.670	C/A	39,59	C/A	52,47	C/A	46,42	C/A	23,19	C/C	5,0	C/C	8,0	A/A	0,5	A/A	0,5
12	18463066	rs332912108	A	G	GFAP	0.613	0.455	0.500	0.500	FALSE	0.207	0.601	1.000	1.000	A/G	18,15	A/G	12,19	A/G	15,15	A/G	11,11	G/G	0,6	G/G	0,6	A/A	4,0	A/A	3,0
12	18505889	rs331027231	G	A	EFTUD2	1.000	0.615	0.500	0.467	FALSE	0.000	0.403	1.000	0.796	G/A	8,5	G/G	22,0	G/A	8,7	G/A	4,4	G/G	6,0	G/G	5,0	A/A	0,4	A/A	0,3
12	19266910	rs340982143	A	G	MPP2	0.552	0.500	0.652	0.519	FALSE	0.577	1.000	0.141	0.847	A/G	14,14	A/G	16,13	A/G	13,14	A/G	8,15	A/A	4,0	A/A	10,0	G/G	0,5	G/G	0,5
12	26903915	rs325503261	C	T	CACNA1G	0.714	0.429	0.000	0.222	FALSE	0.103	0.705	0.019	0.086	C/T	3,4	C/T	10,4	C/T	7,2	C/C	4,0	C/C	6,0	C/C	3,0	T/T	0,5	T/T	0,4
12	31641215	rs335471107	T	C	HLF	0.730	0.654	0.500	0.425	FALSE	0.004	0.114	1.000	0.342	T/C	17,9	T/C	27,10	T/C	17,23	T/C	14,14	C/C	0,3	T/T	6,0	C/C	0,3	T/T	4,0
12	38022517	rs330803301	G	A	ZNHIT3	0.444	0.556	0.889	1.000	FALSE	0.739	0.739	0.013	0.000	G/A	5,4	G/A	4,5	A/A	0,13	G/A	1,8	G/G	5,0	G/G	3,0	A/A	0,5	A/A	0,7
12	38583350	rs81303285	G	A	ACACA	0.619	0.357	0.417	0.261	FALSE	0.273	0.128	0.563	0.019	G/A	10,18	G/A	13,8	G/A	17,6	G/A	7,5	G/G	3,0	G/G	6,0	A/A	0,4	A/A	0,7
12	38583468	rs81303284	T	A	ACACA	0.379	0.450	0.000	0.474	FALSE	0.191	0.654	0.002	0.819	T/A	9,11	T/A	11,18	T/A	10,9	T/T	7,0	T/T	5,0	T/T	3,0	A/A	0,4	A/A	0,6
12	39766233	.	G	T	MMP28	1.000	0.714	0.333	0.444	FALSE	0.041	0.249	0.410	0.739	G/T	5,2	G/G	3,0	G/T	5,4	G/T	4,2	G/G	3,0	G/G	9,0	T/T	0,3	T/T	0,4
12	40040239	rs320712471	G	A	NLE1	0.667	0.000	0.000	0.000	FALSE	0.560	0.001	0.004	0.002	A/A	0,8	G/A	2,1	G/G	7,0	G/G	6,0	G/G	3,0	G/G	3,0	A/A	0,6	A/A	0,5
12	42087837	rs340684704	G	T	TMEM98	0.400	0.200	0.733	0.750	FALSE	0.654	0.165	0.065	0.076	G/T	1,4	G/T	2,3	G/T	3,9	G/T	4,11	G/G	7,0	G/G	4,0	T/T	0,5	T/T	0,3
12	42088537	rs691250984	C	T	TMEM98	0.222	0.222	0.375	0.545	FALSE	0.086	0.086	0.477	0.763	C/T	2,7	C/T	2,7	C/T	5,6	C/T	5,3	C/C	3,0	C/C	4,0	T/T	0,3	T/T	0,3
12	42576589	rs338433700	T	C	ZNF207	0.500	0.429	0.429	0.250	FALSE	1.000	0.705	0.705	0.306	T/C	4,3	T/C	2,2	T/C	1,3	T/C	3,4	C/C	0,3	C/C	0,3	T/T	5,0	T/T	5,0
12	42623349	rs338873328	C	A	C17orf75	0.556	0.556	0.000	0.571	FALSE	0.739	0.739	0.041	0.705	C/A	5,4	C/A	5,4	C/A	3,4	C/C	3,0	C/C	3,0	C/C	7,0	A/A	0,10	A/A	0,7
12	42623376	rs318662914	G	A	C17orf75	0.500	0.625	0.333	0.600	FALSE	1.000	0.477	0.560	0.654	G/A	3,5	G/A	4,4	G/A	3,2	G/A	1,2	A/A	0,4	A/A	0,5	G/G	7,0	G/G	6,0
12	42624043	rs333070311	T	C	C17orf75	0.526	0.750	0.500	0.800	FALSE	0.819	0.076	1.000	0.050	T/C	9,3	T/C	10,9	T/C	2,8	T/C	2,2	T/T	5,0	T/T	5,0	C/C	0,7	C/C	0,4
12	42624462	rs328251095	T	C	C17orf75	0.417	0.533	1.000	0.500	FALSE	0.563	0.796	0.019	1.000	T/C	7,8	T/C	7,5	T/C	4,4	T/T	4,0	C/C	0,3	C/C	0,6	T/T	3,0	T/T	6,0
12	42624894	rs323905405	G	A	C17orf75	0.375	0.600	0.667	0.667	FALSE	0.315	0.654	0.313	0.192	G/A	2,3	G/A	10,6	G/A	10,5	G/A	6,3	A/A	0,6	A/A	0,3	G/G	6,0	G/G	5,0
12	42624897	rs335720621	T	G	C17orf75	0.375	0.571	0.600	0.533	FALSE	0.315	0.705	0.526	0.796	T/G	4,3	T/G	6,10	T/G	7,8	T/G	4,6	T/T	6,0	T/T	5,0	G/G	0,5	G/G	0,5
12	43898835	rs321053477	A	G	WSB1	0.333	0.632	0.750	0.333	FALSE	0.123	0.249	0.306	0.410	A/G	12,7	A/G	7,14	A/G	4,2	A/G	1,3	A/A	6,0	A/A	3,0	G/G	0,3	G/G	0,7
12	43903782	rs342861187	T	C	WSB1	0.476	0.481	0.622	0.510	FALSE	0.758	0.847	0.099	0.886	T/C	13,14	T/C	20,22	T/C	24,25	T/C	17,28	T/T	6,0	T/T	3,0	C/C	0,6	C/C	0,4
12	44609193	rs332844684	A	T	TMEM97	0.478	0.308	0.517	0.400	FALSE	0.835	0.160	0.853	0.272	A/T	4,9	A/T	11,12	A/T	18,12	A/T	14,15	A/A	5,0	A/A	4,0	T/T	0,3	T/T	0,3
12	44609288	rs331287514	G	A	TMEM97	0.412	0.357	0.381	0.565	FALSE	0.466	0.282	0.273	0.531	G/A	9,5	G/A	10,7	G/A	13,10	G/A	8,13	A/A	0,6	A/A	0,5	G/G	6,0	G/G	5,0
12	44609326	rs322278716	C	T	TMEM97	0.609	0.500	0.550	0.679	FALSE	0.295	1.000	0.654	0.056	C/T	7,7	C/T	9,14	C/T	19,9	C/T	11,9	T/T	0,7	T/T	0,4	C/C	3,0	C/C	4,0
12	44609659	rs343405617	G	A	TMEM97	0.400	0.667	0.375	0.438	FALSE	0.526	0.313	0.477	0.617	G/A	3,6	G/A	6,4	G/A	7,9	G/A	3,5	A/A	0,3	A/A	0,7	G/G	6,0	G/G	3,0
12	44624874	rs336980352	G	A	TNFAIP1	0.333	0.364	0.400	0.615	FALSE	0.244	0.363	0.654	0.403	G/A	7,4	G/A	8,4	G/A	8,5	G/A	2,3	A/A	0,3	A/A	0,4	G/G	3,0	G/G	4,0
12	44640940	rs327335136	C	G	TMEM199	0.667	0.500	0.565	0.360	FALSE	0.192	1.000	0.531	0.159	C/G	9,9	C/G	5,10	C/G	9,16	C/G	13,10	G/G	0,4	G/G	0,9	C/C	5,0	C/C	3,0
12	44801841	rs81318716	A	G	UNC119	0.583	0.000	0.429	0.286	FALSE	0.563	0.019	0.512	0.046	G/G	0,4	A/G	7,5	A/G	15,6	A/G	12,9	A/A	4,0	A/A	3,0	G/G	0,3	G/G	0,4
12	44901919	rs81212845	G	A	SDF2	0.640	0.440	0.571	0.500	FALSE	0.159	0.548	0.705	1.000	G/A	11,14	G/A	16,9	G/A	12,12	G/A	3,4	G/G	3,0	G/G	8,0	A/A	0,5	A/A	0,5
12	44979758	rs323207850	C	T	TRAF4	0.800	0.000	0.667	1.000	FALSE	0.165	0.019	0.410	0.002	C/C	4,0	C/T	1,4	C/C	7,0	C/T	4,2	T/T	0,3	T/T	0,4	C/C	3,0	C/C	3,0

12	44987587	rs328692439	G	A	FAM222B	0.533	0.583	0.714	0.467	FALSE	0.796	0.563	0.249	0.796	G/A	7,5	G/A	8,7	G/A	8,7	G/A	2,5	G/G	4,0	G/G	3,0	A/A	0,4	A/A	0,4
12	45333727	rs322589511	A	C	ENSSSCG00000017783	0.568	0.630	0.667	1.000	FALSE	0.365	0.175	0.065	0.000	A/C	17,10	A/C	25,19	C/C	0,43	A/C	10,20	A/A	3,0	A/A	5,0	C/C	0,4	C/C	0,3
12	45357291	rs329422907	A	G	ENSSSCG00000017783	0.525	0.667	0.000	0.400	FALSE	0.752	0.043	0.004	0.369	A/G	24,12	A/G	21,19	A/G	12,8	A/A	6,0	A/A	5,0	A/A	4,0	G/G	0,5	G/G	0,3
12	46343151	rs338738147	A	G	ENSSSCG00000017796	0.476	0.500	0.600	1.000	FALSE	0.827	1.000	0.369	0.000	A/G	10,10	A/G	10,11	G/G	0,22	A/G	8,12	A/A	3,0	A/A	7,0	G/G	0,3	G/G	0,4
12	46820912	rs346331909	G	A	ABR	0.500	0.535	0.600	0.750	FALSE	1.000	0.647	0.654	0.041	G/A	23,20	G/A	20,20	G/A	4,12	G/A	2,3	G/G	3,0	G/G	4,0	A/A	0,5	A/A	0,6
12	46828749	rs336851048	G	C	ABR	0.385	0.567	0.000	0.632	FALSE	0.095	0.301	0.004	0.249	G/C	34,26	G/C	20,32	G/C	7,12	G/G	6,0	G/G	4,0	G/G	4,0	C/C	0,3	C/C	0,3
12	46854049	rs332455497	T	C	ABR	0.529	0.592	0.625	0.444	FALSE	0.628	0.122	0.315	0.505	T/C	42,29	T/C	36,32	T/C	20,16	T/C	6,10	T/T	4,0	T/T	3,0	C/C	0,5	C/C	0,3
12	46894638	rs318247815	C	T	ABR	0.314	0.403	0.558	0.560	FALSE	0.002	0.126	0.405	0.298	C/T	25,37	C/T	22,48	C/T	33,42	C/T	23,29	C/C	3,0	C/C	3,0	T/T	0,4	T/T	0,4
12	47719289	rs329700847	C	T	INPP5K	0.500	0.667	0.000	1.000	FALSE	1.000	0.410	0.041	0.004	C/T	4,2	C/T	4,4	T/T	0,6	C/C	3,0	C/C	3,0	C/C	10,0	T/T	0,4	T/T	0,5
12	48734215	rs336257953	T	G	PAFAH1B1	0.000	0.549	0.440	0.426	FALSE	0.000	0.406	0.548	0.248	T/G	39,32	G/G	0,66	T/G	35,26	T/G	14,11	T/T	3,0	T/T	3,0	G/G	0,4	G/G	0,5
12	51157709	.	G	A	WSCD1	0.600	0.818	0.667	0.800	FALSE	0.369	0.028	0.560	0.165	G/A	9,2	G/A	12,8	G/A	1,4	G/A	1,2	G/G	4,0	G/G	4,0	A/A	0,3	A/A	0,3
12	51164406	.	C	A	WSCD1	0.543	0.550	0.500	0.455	FALSE	0.555	0.654	1.000	0.670	C/A	11,9	C/A	25,21	C/A	12,10	C/A	7,7	C/C	5,0	C/C	3,0	A/A	0,5	A/A	0,4
12	51505667	.	G	A	MIS12	0.500	0.375	0.500	0.400	FALSE	1.000	0.477	1.000	0.526	G/A	3,5	G/A	5,5	G/A	6,4	G/A	6,6	G/G	3,0	G/G	3,0	A/A	0,6	A/A	0,3
12	51602995	.	C	G	NUP88	0.370	0.371	0.529	0.514	FALSE	0.175	0.126	0.732	0.869	C/G	22,13	C/G	17,10	C/G	19,18	C/G	18,16	G/G	0,3	G/G	0,4	C/C	5,0	C/C	4,0
12	52074358	.	C	T	PLD2	0.000	0.600	0.571	0.200	FALSE	0.019	0.654	0.705	0.016	C/T	3,2	T/T	0,4	C/T	12,3	C/T	3,4	C/C	3,0	C/C	3,0	T/T	0,4	T/T	0,3
12	52432849	rs339974843	T	G	BCL6B	0.167	0.500	0.556	0.625	FALSE	0.088	1.000	0.739	0.477	T/G	2,2	T/G	1,5	T/G	3,5	T/G	4,5	T/T	3,0	T/T	3,0	G/G	0,3	G/G	0,5
12	52439106	rs1111479597	C	T	SLC16A13	1.000	0.625	0.000	0.000	FALSE	0.019	0.477	0.019	0.008	C/T	5,3	C/C	4,0	C/C	5,0	C/C	4,0	C/C	7,0	C/C	4,0	T/T	0,5	T/T	0,4
12	52671934	rs81436801	C	A	NEURL4	0.571	0.571	0.400	0.545	FALSE	0.592	0.592	0.526	0.670	C/A	8,6	C/A	8,6	C/A	10,12	C/A	6,4	C/C	4,0	C/C	6,0	A/A	0,3	A/A	0,6
12	52719521	rs340418192	G	C	TNK1	0.250	0.444	0.385	0.588	FALSE	0.041	0.739	0.403	0.466	G/C	4,5	G/C	4,12	G/C	7,10	G/C	8,5	G/G	3,0	G/G	3,0	C/C	0,3	C/C	0,5
12	52929308	rs330866136	G	C	ATP1B2	0.538	0.553	0.692	0.510	FALSE	0.502	0.358	0.047	0.886	G/C	42,34	G/C	43,37	G/C	24,25	G/C	8,18	G/G	4,0	G/G	4,0	C/C	0,3	C/C	0,3
12	53176146	rs330941982	G	A	KCNAB3	0.500	0.778	0.000	0.500	FALSE	1.000	0.086	0.008	1.000	G/A	7,2	G/A	5,5	G/A	3,3	G/G	5,0	G/G	5,0	G/G	4,0	A/A	0,5	A/A	0,3
12	53548355	rs342547162	T	C	ENSSSCG00000028850	0.720	0.500	0.258	0.333	FALSE	0.025	1.000	0.006	0.080	T/C	9,9	T/C	18,7	T/C	18,9	T/C	23,8	T/T	6,0	T/T	8,0	C/C	0,5	C/C	0,4
12	55408591	rs698818034	C	G	SCO1	0.444	0.700	0.500	0.400	FALSE	0.739	0.200	1.000	0.654	C/G	7,3	C/G	4,5	C/G	3,2	C/G	4,4	C/C	8,0	C/C	6,0	G/G	0,6	G/G	0,4
13	23833737	rs321830568	T	C	CSRNP1	0.333	0.200	1.000	0.800	FALSE	0.410	0.165	0.041	0.165	T/C	1,4	T/C	2,4	T/C	1,4	C/C	0,3	T/T	5,0	T/T	4,0	C/C	0,6	C/C	0,3
13	24039485	rs323773954	T	C	SLC25A38	0.462	0.529	0.667	0.600	FALSE	0.781	0.808	0.244	0.526	T/C	9,8	T/C	6,7	T/C	4,6	T/C	4,8	T/T	5,0	T/T	4,0	C/C	0,4	C/C	0,3
13	28691887	rs340334178	C	A	LARS2	0.444	0.667	0.400	0.375	FALSE	0.739	0.410	0.654	0.477	C/A	4,2	C/A	4,5	C/A	5,3	C/A	3,2	C/C	8,0	C/C	4,0	A/A	0,3	A/A	0,4
13	28810431	rs325121696	G	A	LIMD1	0.455	0.500	0.500	0.500	FALSE	0.763	1.000	1.000	1.000	G/A	3,3	G/A	6,5	G/A	4,4	G/A	4,4	A/A	0,4	A/A	0,3	G/G	7,0	G/G	3,0
13	29874356	.	G	A	NBEAL2	0.333	0.375	0.400	0.833	FALSE	0.313	0.477	0.654	0.088	G/A	3,5	G/A	3,6	G/A	1,5	G/A	3,2	G/G	3,0	G/G	5,0	A/A	0,4	A/A	0,4
13	45164072	rs346396594	G	T	SYNPR	0.467	0.545	0.526	0.480	FALSE	0.715	0.670	0.819	0.841	G/T	12,10	G/T	14,16	G/T	13,12	G/T	9,10	G/G	4,0	G/G	6,0	T/T	0,4	T/T	0,7
13	70428580	rs196956667	G	T	CHCHD4	0.714	0.444	0.765	0.500	FALSE	0.103	0.739	0.025	1.000	G/T	4,5	G/T	10,4	G/T	10,10	G/T	4,13	G/G	4,0	G/G	6,0	T/T	0,5	T/T	0,4
13	106609588	rs331719430	C	G	SERPINI1	0.552	0.667	0.571	0.417	FALSE	0.577	0.065	0.705	0.563	C/G	10,20	C/G	13,16	C/G	5,7	C/G	4,3	G/G	0,4	G/G	0,4	C/C	3,0	C/C	4,0
13	117441405	rs695645881	G	A	MFN1	0.429	0.474	0.516	0.583	FALSE	0.512	0.819	0.857	0.413	G/A	10,9	G/A	12,9	G/A	14,10	G/A	16,15	A/A	0,6	A/A	0,5	G/G	8,0	G/G	5,0
13	117446434	rs1107426214	G	C	GNB4	0.500	0.579	1.000	1.000	FALSE	1.000	0.490	0.041	0.041	G/C	8,11	G/C	10,10	G/G	3,0	G/G	3,0	C/C	0,5	C/C	0,6	G/G	3,0	G/G	3,0
13	118627095	rs333749997	A	G	ENSSSCG00000011768	0.500	0.800	0.333	0.333	FALSE	1.000	0.165	0.560	0.560	A/G	1,4	A/G	5,5	A/G	1,2	A/G	1,2	G/G	0,6	G/G	0,4	A/A	5,0	A/A	6,0
13	132274335	rs320782025	A	G	ACAP2	0.444	0.400	0.385	0.500	FALSE	0.739	0.654	0.403	1.000	A/G	3,2	A/G	5,4	A/G	7,7	A/G	5,8	G/G	0,6	G/G	0,6	A/A	9,0	A/A	7,0
13	135613360	rs334992997	G	C	UMPS	0.385	0.000	1.000	0.750	FALSE	0.403	0.000	0.008	0.306	G/G	14,0	G/C	8,5	G/C	3,1	G/G	5,0	C/C	0,8	C/C	0,3	G/G	6,0	G/G	6,0
13	135613368	rs342940692	G	A	UMPS	0.385	0.000	1.000	0.333	FALSE	0.403	0.000	0.019	0.560	G/G	14,0	G/A	8,5	G/A	1,2	G/G	4,0	A/A	0,8	A/A	0,3	G/G	4,0	G/G	6,0
13	138102929	rs339714456	A	G	KPNA1	0.313	0.286	0.000	0.250	FALSE	0.129	0.249	0.008	0.148	A/G	5,2	A/G	11,5	A/G	2,6	G/G	0,5	G/G	0,5	G/G	0,3	A/A	5,0	A/A	6,0
13	140729496	.	C	A	CD80	0.333	0.571	0.857	1.000	FALSE	0.313	0.592	0.047	0.000	C/A	8,6	C/A	3,6	A/A	0,22	C/A	1,6	C/C	5,0	C/C	5,0	A/A	0,6	A/A	0,5
13	182185856	rs324885873	C	T	ENSSSCG00000032702	0.533	0.455	0.286	0.385	FALSE	0.796	0.763	0.249	0.403	C/T	5,6	C/T	8,7	C/T	8,5	C/T	5,2	C/C	3,0	C/C	5,0	T/T	0,5	T/T	0,3
13	202483079	.	G	A	ETS2	0.727	0.615	0.917	0.800	FALSE	0.125	0.403	0.002	0.016	G/A	8,5	G/A	8,3	G/A	3,12	G/A	1,11	G/G	3,0	G/G	8,0	A/A	0,3	A/A	0,4
14	10344086	rs330705168	C	T	BNIP3L	0.533	0.640	0.818	0.318	FALSE	0.715	0.159	0.028	0.084	C/T	16,9	C/T	16,14	C/T	15,7	C/T	2,9	C/C	3,0	C/C	6,0	T/T	0,4	T/T	0,7
14	16657547	rs81211097	A	G	SCRG1	0.533	0.613	0.343	0.343	FALSE	0.655	0.207	0.010	0.010	A/G	19,12	A/G	24,21	A/G	44,23	A/G	44,23	A/A	3,0	A/A	5,0	G/G	0,4	G/G	0,7
14	22768716	rs792323573	A	C	ANKLE2	1.000	0.250	0.000	0.500	FALSE	0.004	0.148	0.041	1.000	A/C	2,6	A/A	6,0	A/C	3,3	A/A	3,0	A/A	3,0	A/A	5,0	C/C	0,4	C/C	0,6
14	22771193	rs1113287171	G	A	ANKLE2	0.400	0.188	0.429	0.467	FALSE	0.369	0.009	0.512	0.796	G/A	3,13	G/A	8,12	G/A	8,7	G/A	12,9	G/G	3,0	G/G	7,0	A/A	0,3	A/A	0,3
14	22771258	rs1107738933	C	A	ANKLE2	0.316	0.333	0.480	0.684	FALSE	0.104	0.192	0.841	0.104	C/A	5,10	C/A	6,13	C/A	6,13	C/A	13,12	C/C	4,0	C/C	7,0	A/A	0,5	A/A	0,8
14	22771260	rs1111856794	C	G	ANKLE2	0.316	0.333	0.480	0.684	FALSE	0.104	0.192	0.841	0.104	C/G	5,10	C/G	6,13	C/G	6,13	C/G	13,12	C/C	6,0	C/C	7,0	G/G	0,5	G/G	0,9
14	24378596	rs320699347	C	T	STX2	0.455	0.571	0.000	0.300	FALSE	0.763	0.705	0.004	0.200	C/T	4,3	C/T	5,6	C/T	7,3	C/C	6,0	C/C	5,0	C/C	5,0	T/T	0,5	T/T	0,6
14	24379011	rs81261040	A	G	STX2	0.400	0.455	0.500	0.429	FALSE	0.654	0.763	1.000	0.705	A/G	6,5	A/G	3,2	A/G	3,4	A/G	6,6	G/G	0,4	G/G	0,5	A/A	4,0	A/A	5,0
14	24379748	rs319544829	C	A	STX2	0.500	1.000	0.400	0.000	FALSE	1.000	0.019	0.654	0.004	C/C	4,0	C/A	4,4	C/C	6,0	C/A	3,2	C/C	12,0	C/C	5,0	A/A	0,3	A/A	0,3
14	24689486	rs80925195	G	A	RIMBP2	0.300	0.438	0.333	1.000	FALSE	0.200	0.617	0.560	0.008	G/A	9,7	G/A	7,3	G/G	5,0	G/A	1,2	A/A	0,4	A/A	0,3	G/G	6,0	G/G	5,0
14	29093293	rs346147949	G	C	CCDC92	0.625	0.484	0.600	0.500	FALSE	0.112	0.857	0.272	1.000	G/C	16,15	G/C	15,25	G/C	18,18	G/C	18,12	C/C	0,4	C/C	0,3	G/G	3,0	G/G	3,0
14	55632959	rs80929479	T	G	LYST	0.231	0.167	0.429	0.286	FALSE	0.046	0.088	0.705	0.249	T/G	1,5	T/G	3,10	T/G	5,2	T/G	4,3	T/T	3,0	T/T	3,0	G/G	0,3	G/G	0,4
14	55633070	rs80997259	A	G	LYST	0.500	0.750	0.400	0.500	FALSE	1.000	0.148	0.654	1.000	A/G	6,2	A/G	5,5	A/G	3,3	A/G	3,2	A/A	10,0	A/A	6,0	G/G	0,5	G/G	0,5
14	55633092	rs80918931	T	C	LYST	0.750	0.714	0.200	0.444	FALSE	0.148	0.249	0.165	0.739	T/C	5,2	T/C	6,2	T/C	5,4	T/C	4,1	T/T	6,0	T/T	6,0	C/C	0,4	C/C	0,4
14	55633333	rs80791649	A	T	LYST	0.600	1.000	0.000	0.538	FALSE	0.654	0.002	0.019	0.781	A/A	7,0	A/T	3,2	A/T	6,7	A/A	4,0	A/A	3,0	A/A	5,0	T/T	0,3	T/T	0,3
14	55633334	rs80921249	G	A	LYST	0.600	1.000	0.000	0.538	FALSE	0.654	0.002	0.019	0.781	G/G	7,0	G/A	3,2	G/A	6,7	G/G	4,0	G/G	4,0	G/G	6,0	A/A	0,4	A/A	0,3
14	74939136	rs328362806	A	G	SPOCK2	0.461	0.437	0.250	0.351	FALSE	0.458	0.238	0.000	0.004	A/G	49,38	A/G	48,41	A/G	33,61	A/G	16,48	G/G	0,4	G/G	0,3	A/A	5,0	A/A	4,0
14	109046026	rs80915187	C	T	AVPI1	0.304	0.455	0.385	0.222	FALSE	0.057	0.763	0.403	0.015	C/T	5,6	C/T	7,16	C/T	14,4	C/T	8,5	C/C	3,0	C/C	3,0	T/T	0,5	T/T	0,4
14	114517531	rs318264182	A	G	NEURL1	0.633	0.478	0.286	0.056	FALSE	0.142	0.835	0.249	0.000	A/G	12,11	A/G	11,19	G/G	1,17	A/G	2,5	G/G	0,4	G/G	0,3	A/A	5,0	A/A	3,0
14	122863897	rs702445549	C	T	VTI1A	0.429	0.533	0.000	0.500	FALSE	0.592	0.796	0.019	1.000	C/T	8,7	C/T	6,8	C/T	2,2	C/C	4,0	C/C	3,0	C/C	6,0	T/T	0,5	T/T	0,5
14	132103321	rs326196958	G	A	ENSSSCG00000010703	0.667	0.765	0.282	0.188	TRUE	0.004	0.000	0.000	0.000	G/A	39,12	G/A	48,24	G/A	69,16	G/A	56,22	G/G	9,0	G/G	4,0	A/A	0,5	A/A	0,3
14	132495049	rs319107261	A	C	ENSSSCG00000010719	0.778	0.920	0.174	0.200	TRUE	0.015	0.000	0.001	0.002	C/C	2,23	A/C	4,14	A/C	5,20	A/C	4,19	C/C	0,4	C/C	0,3	A/A	3,0	A/A	3,0
14	133195744	rs321911821	C	T	GPR26	0.533	0.486	0.346	0.390	FALSE	0.655	0.869	0.114	0.158	C/T	18,19	C/T	24,21	C/T	25,16	C/T	17,9	C/C	5,0	C/C	4,0	T/T	0,5	T/T	0,4
14	133808361	rs325424239	A	G	ENSSSCG00000010734	0.714	0.625	0.171	0.229	FALSE	0.046	0.315	0.000	0.001	A/G	10,6	A/G	15,6	A/G	27,8	A/G	29,6	A/A	6,0	A/A	7,0	G/G	0,4	G/G	0,3
14	133808539	rs324347198	T	C	ENSSSCG00000010734	0.459	0.552	0.548	0.508	FALSE	0.485	0.430	0.412	0.900	T/C	32,26	T/C	34,40	T/C	31,32	T/C	33,40	T/T	5,0	T/T	3,0	C/C	0,4	C/C	0,8

14	135085195	rs331955793	A	G	EDRF1	0.524	0.538	0.556	0.522	FALSE	0.827	0.781	0.637	0.835	A/G	6,7	A/G	10,11	A/G	12,11	A/G	10,8	G/G	0,3	G/G	0,4	A/A	10,0	A/A	7,0
14	135158862	rs327634293	C	T	DHX32	0.700	0.500	0.600	0.000	FALSE	0.200	1.000	0.654	0.002	C/T	5,5	C/T	7,3	C/C	7,0	C/T	2,3	C/C	3,0	C/C	10,0	T/T	0,6	T/T	0,4
14	140503513	rs81450268	T	C	DPYSL4	0.464	0.619	0.250	0.286	FALSE	0.705	0.273	0.076	0.021	T/C	13,8	T/C	13,15	T/C	20,8	T/C	9,3	T/T	3,0	T/T	4,0	C/C	0,3	C/C	0,4
14	140506824	rs343683980	G	C	DPYSL4	0.556	0.542	0.429	0.417	FALSE	0.563	0.683	0.512	0.413	G/C	13,11	G/C	15,12	G/C	14,10	G/C	12,9	G/G	6,0	G/G	3,0	C/C	0,6	C/C	0,8
14	141340359	rs326153482	T	G	ECHS1	0.444	0.286	0.526	0.733	FALSE	0.739	0.103	0.819	0.065	T/G	10,4	T/G	5,4	T/G	11,4	T/G	10,9	G/G	0,4	G/G	0,5	T/T	4,0	T/T	4,0
15	2580050	rs346181662	T	A	LYPD6	0.667	1.000	0.429	0.824	FALSE	0.192	0.019	0.705	0.005	A/A	0,4	T/A	5,10	T/A	14,3	T/A	3,4	A/A	0,5	A/A	0,3	T/T	5,0	T/T	4,0
15	2580530	rs329975451	C	G	LYPD6	0.429	0.667	0.714	0.500	FALSE	0.592	0.410	0.249	1.000	C/G	4,2	C/G	6,8	C/G	8,8	C/G	2,5	C/C	9,0	C/C	3,0	G/G	0,4	G/G	0,6
15	15941322	.	C	T	MCM6	0.250	0.200	0.500	0.833	FALSE	0.041	0.002	1.000	0.088	C/T	5,20	C/T	4,12	C/T	1,5	C/T	2,2	C/C	4,0	C/C	3,0	T/T	0,4	T/T	0,6
15	45040416	rs323773556	G	A	TRAPPC11	0.500	0.583	0.313	0.556	FALSE	1.000	0.563	0.129	0.637	G/A	5,7	G/A	7,7	G/A	10,8	G/A	5,11	A/A	0,6	A/A	0,6	G/G	8,0	G/G	5,0
15	45040433	rs329801343	A	T	TRAPPC11	0.500	0.444	0.250	0.600	FALSE	1.000	0.739	0.041	0.369	A/T	5,4	A/T	7,7	A/T	12,8	A/T	4,12	T/T	0,8	T/T	0,6	A/A	8,0	A/A	6,0
15	48573615	rs331902409	G	A	BRF2	0.556	0.375	0.333	0.417	FALSE	0.739	0.477	0.410	0.563	G/A	3,5	G/A	5,4	G/A	7,5	G/A	4,2	G/G	5,0	G/G	3,0	A/A	0,7	A/A	0,5
15	48573832	rs342688043	T	C	BRF2	0.692	0.692	0.364	0.615	FALSE	0.160	0.160	0.363	0.403	T/C	9,4	T/C	9,4	T/C	5,8	T/C	7,4	T/T	4,0	T/T	3,0	C/C	0,5	C/C	0,5
15	48644486	rs335902683	G	A	ERLIN2	0.063	0.444	0.000	0.471	FALSE	0.000	0.739	0.008	0.808	G/A	4,5	A/A	1,15	G/A	9,8	G/G	5,0	G/G	3,0	G/G	6,0	A/A	0,8	A/A	0,8
15	54916798	rs345078979	A	G	LEPROTL1	0.100	0.286	0.333	0.000	FALSE	0.007	0.249	0.410	0.008	A/G	2,5	A/G	1,9	A/A	5,0	A/G	4,2	A/A	9,0	A/A	6,0	G/G	0,13	G/G	0,5
15	57044378	rs81234906	C	T	AMER3	0.000	0.286	0.435	0.583	FALSE	0.000	0.249	0.531	0.563	C/T	5,2	C/C	14,0	C/T	7,5	C/T	10,13	T/T	0,4	T/T	0,3	C/C	3,0	C/C	3,0
15	96647553	rs337200818	G	A	TMEFF2	0.500	0.750	0.429	0.458	FALSE	1.000	0.004	0.512	0.683	G/A	24,8	G/A	18,18	G/A	13,11	G/A	12,9	G/G	4,0	G/G	4,0	A/A	0,7	A/A	0,4
15	109883825	.	G	A	ADAM23	0.444	0.455	0.429	1.000	FALSE	0.637	0.670	0.705	0.000	G/A	12,10	G/A	10,8	G/G	14,0	G/A	3,4	A/A	0,4	A/A	0,5	G/G	7,0	G/G	3,0
15	111341202	rs322043395	T	G	ENSSSCG00000023264	0.400	1.000	0.500	0.429	FALSE	0.654	0.041	1.000	0.705	T/T	3,0	T/G	2,3	T/G	4,3	T/G	3,3	T/T	3,0	T/T	6,0	G/G	0,8	G/G	0,4
15	125421210	rs55618692	A	G	SCG2	0.457	0.493	0.404	0.466	FALSE	0.473	0.903	0.164	0.558	A/G	33,34	A/G	32,38	A/G	39,34	A/G	31,21	A/A	3,0	A/A	5,0	G/G	0,4	G/G	0,5
15	129309171	rs320367580	A	G	SPHKAP	0.563	0.533	0.417	0.375	FALSE	0.479	0.715	0.413	0.218	A/G	14,16	A/G	14,18	A/G	9,15	A/G	10,14	G/G	0,6	G/G	0,3	A/A	4,0	A/A	5,0
15	129309802	rs325326857	G	A	SPHKAP	0.368	0.400	0.500	0.600	FALSE	0.249	0.437	1.000	0.526	G/A	9,6	G/A	12,7	G/A	6,4	G/A	2,2	A/A	0,8	A/A	0,7	G/G	6,0	G/G	3,0
15	129309808	rs346042969	A	G	SPHKAP	0.368	0.375	0.333	0.500	FALSE	0.249	0.315	0.560	1.000	A/G	10,6	A/G	12,7	A/G	5,5	A/G	1,2	G/G	0,8	G/G	0,6	A/A	6,0	A/A	3,0
15	130079625	.	C	T	PID1	0.429	0.294	0.412	0.519	FALSE	0.592	0.085	0.466	0.847	C/T	12,5	C/T	8,6	C/T	14,13	C/T	7,10	T/T	0,5	T/T	0,6	C/C	4,0	C/C	4,0
15	130079674	.	A	G	PID1	0.391	0.360	0.435	0.455	FALSE	0.295	0.159	0.531	0.670	A/G	16,9	A/G	14,9	A/G	10,12	A/G	10,13	G/G	0,4	G/G	0,4	A/A	7,0	A/A	5,0
15	139881262	rs324876302	G	A	MTERF4	1.000	0.500	0.000	0.000	FALSE	0.041	1.000	0.008	0.041	G/A	3,3	A/A	0,3	A/A	0,3	A/A	0,5	A/A	0,4	A/A	0,6	G/G	7,0	G/G	3,0
16	22846416	rs324724954	C	G	WDR70	0.917	0.700	0.571	0.600	FALSE	0.002	0.200	0.705	0.654	C/G	3,7	G/G	1,11	C/G	3,2	C/G	4,3	G/G	0,3	G/G	0,3	C/C	5,0	C/C	3,0
17	1118013	rs1109881670	T	C	TRMT9B	0.250	0.667	0.333	0.571	FALSE	0.076	0.560	0.410	0.705	T/C	1,2	T/C	9,3	T/C	3,4	T/C	4,2	T/T	11,0	C/C	0,9	T/T	9,0	C/C	0,4
17	5007860	rs328119143	A	G	ENSSSCG00000036785	0.560	0.625	0.333	0.000	FALSE	0.548	0.218	0.313	0.000	A/G	9,15	A/G	11,14	A/A	13,0	A/G	6,3	A/A	5,0	G/G	0,5	A/A	5,0	G/G	0,3
17	13774603	rs339178229	G	A	RASSF2	0.188	0.500	0.000	0.500	FALSE	0.009	1.000	0.019	1.000	G/A	3,3	G/A	3,13	G/A	4,4	G/G	4,0	G/G	4,0	G/G	5,0	A/A	0,5	A/A	0,3
17	26353618	rs709873554	C	T	ENSSSCG00000007088	0.500	0.364	0.375	0.667	FALSE	1.000	0.363	0.315	0.244	C/T	4,7	C/T	8,8	C/T	4,8	C/T	10,6	C/C	5,0	C/C	6,0	T/T	0,8	T/T	0,7
17	30362261	rs325950129	T	G	NAPB	1.000	0.833	0.527	0.471	FALSE	0.000	0.000	0.642	0.632	T/G	60,12	T/T	54,0	T/G	37,33	T/G	35,39	T/T	3,0	T/T	3,0	G/G	0,6	G/G	0,5
17	36425119	rs319997714	A	T	ENSSSCG00000007253	0.304	0.375	0.500	0.240	FALSE	0.057	0.218	1.000	0.008	A/T	9,15	A/T	7,16	A/T	19,6	A/T	4,4	A/A	5,0	A/A	3,0	T/T	0,3	T/T	0,3
17	36425496	rs80811232	G	A	ENSSSCG00000007253	0.208	0.533	0.238	0.737	FALSE	0.003	0.796	0.014	0.035	G/A	8,7	G/A	5,19	G/A	5,14	G/A	16,5	G/G	4,0	G/G	4,0	A/A	0,4	A/A	0,4
17	48717604	.	C	G	SLC13A3	0.400	0.600	0.400	0.333	FALSE	0.654	0.654	0.654	0.410	C/G	2,3	C/G	3,2	C/G	2,4	C/G	2,3	G/G	0,6	G/G	0,4	C/C	4,0	C/C	3,0
17	49258997	rs325635379	T	C	ENSSSCG00000007454	0.571	0.615	0.579	0.571	FALSE	0.512	0.403	0.490	0.449	T/C	5,8	T/C	9,12	T/C	16,12	T/C	11,8	C/C	0,6	C/C	0,6	T/T	4,0	T/T	4,0
17	58864547	rs80941164	C	T	ENSSSCG00000007516	0.625	0.500	0.579	0.542	FALSE	0.155	1.000	0.490	0.683	C/T	15,15	C/T	20,12	C/T	11,13	C/T	8,11	C/C	5,0	C/C	5,0	T/T	0,10	T/T	0,10
18	1934039	rs324155557	A	G	LMBR1	0.393	0.400	0.500	1.000	FALSE	0.255	0.437	1.000	0.000	A/G	6,9	A/G	11,17	G/G	0,23	A/G	5,5	A/A	5,0	A/A	3,0	G/G	0,4	G/G	0,5
18	6861381	rs55618276	G	A	ENSSSCG00000016459	0.383	0.500	0.464	0.491	FALSE	0.107	1.000	0.705	0.891	G/A	16,16	G/A	29,18	G/A	26,27	G/A	13,15	A/A	0,5	A/A	0,4	G/G	5,0	G/G	4,0
18	6900298	rs333623648	T	C	FAM131B	0.500	0.571	0.563	0.700	FALSE	1.000	0.397	0.617	0.070	T/C	15,20	T/C	19,19	T/C	14,6	T/C	9,7	C/C	0,6	C/C	0,3	T/T	5,0	T/T	7,0
18	6900702	rs328110727	G	C	FAM131B	0.432	0.538	0.435	0.500	FALSE	0.410	0.695	0.531	1.000	G/C	12,14	G/C	21,16	G/C	20,20	G/C	10,13	C/C	0,4	C/C	0,3	G/G	5,0	G/G	5,0
18	6997223	rs321436567	C	T	GSTK1	0.750	0.857	0.500	0.357	FALSE	0.076	0.047	1.000	0.282	C/T	1,6	C/T	3,9	C/T	5,9	C/T	2,2	T/T	0,3	T/T	0,3	C/C	6,0	C/C	3,0
18	8894150	rs339894067	G	T	ENSSSCG00000013470	0.375	0.571	0.250	0.700	FALSE	0.477	0.705	0.306	0.200	G/T	3,4	G/T	5,3	G/T	7,3	G/T	1,3	T/T	0,5	T/T	0,4	G/G	4,0	G/G	6,0
18	9442512	.	T	C	SLC37A3	0.600	0.750	0.600	0.000	FALSE	0.654	0.306	0.526	0.041	T/C	1,3	T/C	2,3	C/C	0,3	T/C	6,4	C/C	0,8	C/C	0,3	T/T	5,0	T/T	3,0
18	11056729	rs703192176	T	C	TRIM24	0.857	0.750	0.500	0.250	FALSE	0.047	0.076	1.000	0.306	T/C	3,9	T/C	1,6	T/C	1,3	T/C	2,2	C/C	0,5	C/C	0,6	T/T	5,0	T/T	3,0
18	15109652	rs343285937	T	C	EXOC4	0.615	0.625	0.500	0.615	FALSE	0.403	0.477	1.000	0.237	T/C	3,5	T/C	5,8	T/C	16,10	T/C	4,4	C/C	0,7	C/C	0,5	T/T	4,0	T/T	5,0
18	15109902	rs345125679	T	G	EXOC4	0.625	0.667	0.500	0.389	FALSE	0.315	0.192	1.000	0.344	T/G	5,10	T/G	6,10	T/G	7,11	T/G	4,4	G/G	0,5	G/G	0,5	T/T	3,0	T/T	7,0
18	18462280	rs323207406	C	A	CEP41	0.857	1.000	0.750	0.286	FALSE	0.047	0.002	0.148	0.249	A/A	0,7	C/A	1,6	C/A	2,5	C/A	6,2	A/A	0,3	A/A	0,4	C/C	4,0	C/C	3,0
18	25038540	rs333055902	A	T	AASS	1.000	0.000	0.000	0.500	FALSE	0.002	0.041	0.041	1.000	A/A	3,0	T/T	0,7	A/T	3,3	T/T	0,3	T/T	0,4	T/T	0,3	A/A	9,0	A/A	5,0
18	25111477	rs81486470	G	A	PTPRZ1	0.529	0.450	0.333	0.000	FALSE	0.808	0.654	0.560	0.041	G/A	11,9	G/A	8,9	A/A	0,3	G/A	1,2	A/A	0,7	A/A	0,3	G/G	3,0	G/G	8,0
18	37648738	rs344445809	C	T	ANLN	0.143	0.200	0.600	0.667	FALSE	0.047	0.165	0.654	0.560	C/T	1,4	C/T	1,6	C/T	1,2	C/T	2,3	C/C	3,0	C/C	4,0	T/T	0,5	T/T	0,3
18	48609801	rs1107572111	C	T	VOPP1	0.750	0.200	1.000	0.818	FALSE	0.148	0.050	0.019	0.028	C/T	8,2	C/T	2,6	C/T	9,2	C/C	4,0	T/T	0,3	T/T	0,3	C/C	3,0	C/C	4,0
18	50961164	rs333701744	G	A	YKT6	0.750	0.318	0.300	0.214	FALSE	0.041	0.084	0.200	0.027	G/A	15,7	G/A	4,12	G/A	3,11	G/A	3,7	A/A	0,3	A/A	0,6	G/G	4,0	G/G	4,0
18	51325592	rs339325940	G	A	ENSSSCG00000016758	0.320	0.000	0.641	0.826	FALSE	0.069	0.000	0.076	0.000	A/A	0,17	G/A	8,17	G/A	8,38	G/A	14,25	G/G	4,0	G/G	4,0	A/A	0,3	A/A	0,4
X	90704542	rs324650266	A	G	CHRDL1	0.000	0.400	0.000	0.625	FALSE	0.000	0.437	0.002	0.315	A/G	9,6	A/A	18,0	A/G	10,6	G/G	0,7	G/G	0,7	G/G	0,3	A/A	13,0	A/A	3,0
AEMK02000452.1	917166	.	T	C	HSP90AA1	0.151	0.510	0.779	0.638	FALSE	0.000	0.845	0.000	0.001	T/C	53,51	T/C	16,90	T/C	50,88	T/C	31,109	T/T	4,0	T/T	3,0	C/C	0,4	C/C	0,3
AEMK02000682.1	417433	.	T	C	ZMYND19	0.600	0.556	0.583	0.722	FALSE	0.437	0.739	0.413	0.055	T/C	4,5	T/C	6,9	T/C	5,13	T/C	10,14	T/T	3,0	C/C	0,3	T/T	4,0	C/C	0,3
